# A prospective analysis of lymph node retrieval in colorectal cancer: discrepancies, neoadjuvant impact, and practical implications

**DOI:** 10.3389/fmed.2025.1611170

**Published:** 2025-10-01

**Authors:** Saumik Biswas, Jesse R. Walsh, Sami Ul Haq, Ann M. Marilley, Elizabeth P. Pasman, Monica Kendall, Matthew Cecchini, Grace Lebbin, Darren Wilson

**Affiliations:** ^1^Department of Pathology and Laboratory Medicine, Schulich School of Medicine and Dentistry, Western University, London, ON, Canada; ^2^Tenomix Inc., London, ON, Canada; ^3^Advanced Diagnostics Laboratory, Mayo Clinic, Rochester, MN, United States; ^4^Department of Laboratory Medicine and Pathology, Mayo Clinic, Rochester, MN, United States

**Keywords:** lymph nodes, manual dissection, colorectal cancer, grossing, pathologists’ assistant, economics

## Abstract

**Introduction:**

Accurate lymph node (LN) retrieval is vital for colorectal cancer (CRC) staging and determining adjuvant therapy.

**Methods:**

In this prospective study of 122 CRC specimens, we evaluated LN retrieval times, discrepancies between gross and microscopic LN counts, and the impact of neoadjuvant therapy.

**Results:**

On average, dissecting each specimen took 50 min (range 15–295 min), with rectal and descending/sigmoid colon specimens often requiring multiple passes. Macroscopic dissection yielded an average of 45.1 LNs per specimen, whereas microscopic examination confirmed only 35.7 LNs on average. Neoadjuvant therapy did not alter macroscopic yields (*p* = 0.105), yet significantly reduced microscopic LN counts (*p* = 2.676 × 10^5^). T-stage correlated with total microscopic LN counts (*p* = 0.018) but not the number of cancer-positive nodes (*p* = 0.140). Rectal specimens showed the largest discrepancy between macroscopic and microscopic LN counts; in contrast, 8 specimens had higher microscopic than macroscopic counts, suggesting that some LNs were missed during manual palpation but detected microscopically in the extra submitted sections of mesenteric tissue. Overall, the mean absolute percentage error (MAPE) was 50.18%, rising to 97.87% for neoadjuvant-treated cases. The average pathology report turnaround time (TAT) was 3.3 business days, meeting the recommended 4-days threshold, with no significant delay due to cancer location or additional LN searches. A preliminary cost analysis indicates that missed or misidentified LNs can increase histology processing and pathologist review expenses, emphasizing the need for more efficient LN search protocols.

**Discussion:**

Taken together, these findings emphasize the multifactorial nature of LN retrieval challenges, particularly in neoadjuvant-treated and anatomically complex cases. Refining dissection protocols, leveraging new technologies, and allocating adequate resources may help reduce retrieval errors, potentially improving staging accuracy and clinical decision-making.

## 1 Introduction

In 2020, colorectal cancer (CRC) was identified as the third most frequently diagnosed cancer worldwide and the second leading cause of cancer-related deaths, with approximately 1.9 million new cases and 930,000 fatalities attributed to this disease (1, 2). The incidence and mortality rates vary significantly across regions, with higher incidences in developed areas and contrasting mortality patterns observed globally. Projections indicate that by 2040, CRC will account for 3.2 million new cases and 1.6 million deaths, predominantly in countries with high Human Development Index scores (2). Moreover, the recent COVID-19 pandemic has disrupted screening programs, and modeling studies suggest that even a 12-months screening interruption could result in 5,212 additional diagnoses and 2,366 extra deaths in Canada between 2020 and 2050 (3). This scenario emphasizes the urgent need for research and interventions to address the escalating global burden of CRC.

Accurate staging of CRC is crucial for determining appropriate treatment plans and prognostication. A key component of staging is the histological evaluation of regional lymph nodes (LNs), with numerous studies demonstrating that LN status and yield are significant predictors of patient survival (4–9). In node-positive disease, adjuvant chemotherapy is generally recommended, while its benefit in stage II cancers is minimal (10), highlighting the importance of identifying additional high-risk features, such as a low LN yield, which may influence treatment decisions (11–16).

Current CAP and AJCC guidelines recommend the examination of at least 12 LNs from CRC resection specimens (17), although experts debate the optimal number required, with suggestions ranging from 9 to over 30 nodes (18–25). The manual LN search process, which involves tactile and visual assessment of excised mesenteric tissues, is laborious and subject to variability based on numerous factors including technical expertise, patient demographics, neoadjuvant therapy, amount of tissue resected, and tumor-specific characteristics (26–34). When fewer than 12 nodes are initially found, a secondary search or adjunct techniques such as fat-clearing may be employed (35). Furthermore, in the United States, it is common practice for Pathologists’ Assistants (PAs) to perform this initial gross examination, with final LN confirmation completed via microscopic evaluation by a pathologist.

Recognizing the inherent limitations of retrospective analyses, we embarked on a prospective, real-time evaluation of the LN search process in 122 colon cancer cases. This first-of-its-kind approach allows for a quantitative assessment of LN retrieval and an exploration of interobserver variability at both the gross and microscopic stages. Furthermore, at the end of our study, we incorporate a high-level economic analysis to evaluate the potential financial implications of current manual LN search practices in pathology laboratories.

## 2 Materials and methods

### 2.1 Patient cohort

Colorectal cancer specimens from 122 patients were collected between June 22 and November 23, 2023. Inclusion criteria required colons having a biopsy-proven diagnosis of invasive adenocarcinoma. Appendiceal carcinomas, anal carcinomas, and other histologic types such as small cell and large cell neuroendocrine carcinoma, mixed adenoneuroendocrine carcinoma, gastrointestinal stromal tumor, and non-gastrointestinal stromal tumor sarcoma were excluded. Local excisions such as transanal disk excisions were also excluded.

### 2.2 Gross examination and lymph node assessment

All included specimens underwent real-time gross dissection during routine clinical workflows, with data actively recorded at the time of examination using predefined documentation templates. Dissection times, mesocolon dimensions, LN sizes, and LN counts were systematically captured prospectively at both the macroscopic and microscopic levels, rather than being retrospectively extracted from pathology reports. This study was approved by the Mayo Clinic Institutional Review Board (IRB) and was designed to assess real-world lymph node retrieval patterns in a forward-looking manner without additional patient enrollment or long-term clinical follow-up.

Prior to gross examination, each patient’s medical record was reviewed for the biopsy diagnosis, imaging studies, and information regarding neoadjuvant therapy. Upon receipt of the colorectal cancer specimen, gross examination was performed according to current CAP and AJCC guidelines (17) for primary carcinoma of the colon and rectum. Measurements included the overall colon length, the three dimensions of the mesenteric tissue, and distances to the nearest mucosal margins, radial/mesenteric margins, and key anatomic landmarks such as the ileocecal valve or dentate line. Tumor stage was later determined by histologic examination.

The tumor or ulcer bed was sectioned and evaluated for the greatest depth of invasion, with most of the sections submitted for frozen evaluation. Subsequently, the mesenteric tissue was carefully removed, palpated, and sectioned to identify all possible LNs. For clarity, throughout this manuscript, the lymph nodes identified during gross examination are referred to as macroscopic LN counts–representing the gross, presumptive LN numbers–while those confirmed on subsequent microscopic evaluation are referred to as microscopic LN counts.

Lymph nodes were processed as formalin-fixed, paraffin-embedded (FFPE) tissues and sectioned at 5-micron thickness for routine hematoxylin and eosin (H&E) staining. Some specimens were initially submitted for frozen section evaluation due to clinical considerations, but all were later processed and reviewed in paraffin to ensure consistency. Immunohistochemistry was not employed, even in ambiguous cases, as the study was designed to reflect routine diagnostic workflows using standard H&E. Furthermore, of note, LNs were not spatially mapped relative to the tumor or anatomical landmarks, nor was tumor–node distance recorded, as mesenteric tissue was removed en bloc prior to node dissection.

In cases where a LN was bisected during sectioning or palpation, it was still counted as a single LN. LNs were then submitted in cassettes containing a maximum of five discrete nodes; if a node was cut, all portions were submitted together and designated as one LN. If fewer than 12 LNs were identified on the initial search, a second examiner performed an additional search. If no further LNs were found, representative sections of mesenteric tissue were submitted for microscopic evaluation, and definitive LN counts were based on the subsequent microscopic confirmation. The maximum dimension (in centimeters) of the smallest and largest LNs was recorded. Additionally, the total time required for mesenteric tissue examination–from the removal of fatty tissue to the final cassette submission–was documented, along with the names of the primary and any secondary grossers.

The majority of gross dissections in our study were performed by PAs, which reflects common practice within the pathology workflow in the United States. Our intent was not to evaluate or compare international practice models or justify the value of PAs, but rather to characterize LN retrieval outcomes as they occur under typical U.S. protocols.

### 2.3 Histopathologic evaluation

Slides were prepared and uploaded to a digital platform (Sectra Digital Pathology Image Management System) for microscopic review. LN identification was performed by board-certified pathologists as part of routine diagnostic workflow. The number of cancer-positive LNs was recorded, with tumor involvement defined as measuring ≥0.2 mm per CAP and AJCC staging criteria. Tumor deposits, defined as invasive adenocarcinoma without any residual LN structure and recorded as N1c, were documented separately. These counts were recorded in the final surgical pathology report and used for comparison with macroscopic LN counts.

### 2.4 Mean absolute percentage error (MAPE) calculations

The Mean Absolute Percentage Error (MAPE) was used to quantify the discrepancy between the predicted (macroscopic) and actual (microscopic) LN counts. MAPE was calculated as the average of the absolute percentage errors–i.e., the absolute difference between the macroscopic and microscopic counts divided by the microscopic count. A lower MAPE indicates closer agreement between estimates and actual counts. For example, if the ground truth is 10 LNs, an estimated count of either 5 or 15 yields a MAPE of 50%, illustrating the metric’s symmetrical nature relative to the ground truth.

### 2.5 Statistical analysis

Statistical analyses were performed using R 4.1.1 (R Core Team, 2021). Outlier and missing data were addressed as follows: a LN_Largest value recorded as 999999 cm was treated as missing, and a tumor stage recorded as “pT4a, pT3” was treated as missing. Outliers in LN counts and LN search time were retained in the data set. After careful review, these values were retained as accurate representations. A sample was defined as processed by a “single grosser” if one examiner performed both the dissection and the screening pass. Business days were defined as Monday through Friday, excluding weekends and six federal holidays.

Differences among subgroups were assessed using either ANOVA or the Kruskal-Wallis non-parametric test based on the extent to which assumptions were met for the respective test. ANOVA compares group means and is interpreted as indicating if there is a difference across group means. Kruskal-Wallis compares differences in the rank sums of the groups and is interpreted as indicating if there is a difference in group distributions. For significance testing using either ANOVA or Kruskal-Wallis where cancer location was included, only samples from “rectum,” “sigmoid colon,” “rectosigmoid colon,” “right/ascending colon,” “cecum,” “transverse colon,” “hepatic flexure,” and “splenic flexure” were included; samples with low counts (e.g., “ileocecal valve,” “left/descending colon,” “descending colon and sigmoid colon,” and “rectum and transverse colon”) were excluded. A pairwise Wilcox test with the p.adjust.method set to “BH” (Benjamini and Hochberg) was used for multiple comparison corrections, and inexact *p*-values were calculated by setting the exact parameter to “FALSE” due to ties in the data.

## 3 Results

### 3.1 Patient cohort

Gross tissue specimens were examined from 122 patients. The majority of samples were from the rectum (29/122), followed by sigmoid colon (24/122), rectosigmoid colon (20/122), collectively comprising 60% of the samples. The remaining samples were from right/ascending colon (18/122), cecum (12/122), transverse colon (5/122), hepatic flexure (4/122), splenic flexure (4/122), ileocecal flexure (2/122), left/descending colon (2/122), rectum and transverse (1/122), and lastly descending and sigmoid colon (1/122). In terms of neoadjuvant status, 63% of patients were not receiving neoadjuvant chemotherapy (77/122), 35% were receiving neoadjuvant chemotherapy (43/122) and 2 had unknown neoadjuvant status. A general overview of the sample characteristics can be found in Table 1.

**TABLE 1:** All study variables examined.

	Rectum	Sigmoid colon	Rectosigmoid colon	Right/ ascending colon	Cecum	Transverse colon	Hepatic flexure	Splenic flexure	Ileocecal valve	Left/ descending colon	Descending colon and sigmoid colon	Rectum and transverse colon	Overall
	(*N* = 29)	(*N* = 24)	(*N* = 20)	(*N* = 18)	(*N* = 12)	(*N* = 5)	(*N* = 4)	(*N* = 4)	(*N* = 2)	(*N* = 2)	(*N* = 1)	(*N* = 1)	(*N* = 122)
**Macro LNs (count)**													
Mean (SD)	44.2 (33.6)	44.5 (16.1)	39.1 (23.3)	51.4 (25.8)	35.7 (18.2)	49.6 (22.1)	43.0 (5.5)	33.5 (7.6)	43.0 (24.0)	28.0 (5.7)	215.0 (NA)	109.0 (NA)	45.1 (29.1)
Median (min, max)	35.0 (14.0, 194.0)	45.0 (17.0, 76.0)	31.5 (13.0, 114.0)	48.0 (18.0, 141.0)	37.0 (11.0, 68.0)	48.0 (23.0, 76.0)	43.0 (37.0, 49.0)	32.5 (26.0, 43.0)	43.0 (26.0, 60.0)	28.0 (24.0, 32.0)	215.0 (215.0, 215.0)	109.0 (109.0, 109.0)	41.0 (11.0, 215.0)
**Micro LNs (count)**													
Mean (SD)	27.6 (29.9)	38.3 (16.8)	25.5 (14.4)	46.6 (26.2)	31.7 (16.7)	40.8 (15.1)	40.0 (4.5)	30.3 (9.7)	39.5 (19.1)	26.0 (2.8)	190.0 (NA)	100.0 (NA)	35.7 (26.8)
Median (min, max)	19.0 (6.0, 166.0)	35.0 (13.0, 72.0)	23.0 (9.0, 67.0)	45.5 (14.0, 136.0)	30.0 (13.0, 65.0)	41.0 (18.0, 58.0)	39.0 (36.0, 46.0)	30.5 (19.0, 41.0)	39.5 (26.0, 53.0)	26.0 (24.0, 28.0)	190.0 (190.0, 190.0)	100.0 (100.0, 100.0)	32.0 (6.0, 190.0)
**Positive LNs (count)**													
Mean (SD)	0.6 (1.7)	0.8 (1.2)	0.3 (0.6)	0.4 (0.8)	2.6 (4.9)	0.4 (0.9)	0.0 (0.0)	1.8 (2.9)	0.0 (0.0)	0.0 (0.0)	0.0 (NA)	0.0 (NA)	0.7 (2.0)
Median (min, max)	0.0 (0.0, 9.0)	0.0 (0.0, 4.0)	0.0 (0.0, 2.0)	0.0 (0.0, 3.0)	0.5 (0.0, 17.0)	0.0 (0.0, 2.0)	0.0 (0.0, 0.0)	0.5 (0.0, 6.0)	0.0 (0.0, 0.0)	0.0 (0.0, 0.0)	0.0 (0.0, 0.0)	0.0 (0.0, 0.0)	0.0 (0.0, 17.0)
**Smallest LN (cm)**													
Mean (SD)	0.2 (0.1)	0.2 (0.2)	0.2 (0.1)	0.2 (0.1)	0.2 (0.1)	0.2 (0.0)	0.2 (0.1)	0.2 (0.1)	0.3 (0.1)	0.2 (0.0)	0.2 (NA)	0.2 (NA)	0.2 (0.1)
Median (min, max)	0.2 (0.1, 0.3)	0.2 (0.1, 0.9)	0.2 (0.1, 0.3)	0.2 (0.1, 0.3)	0.2 (0.1, 0.4)	0.2 (0.2, 0.2)	0.2 (0.1, 0.2)	0.2 (0.1, 0.2)	0.3 (0.2, 0.3)	0.2 (0.2, 0.2)	0.2 (0.2, 0.2)	0.2 (0.2, 0.2)	0.2 (0.1, 0.9)
**Largest LN (cm)**													
Mean (SD)	1.0 (0.5)	1.0 (0.3)	0.9 (0.3)	1.4 (0.6)	2.0 (1.2)	1.2 (0.4)	1.4 (0.2)	1.4 (0.9)	1.2 (0.6)	0.9 (0.2)	3.8 (NA)	1.7 (NA)	1.2 (0.7)
Median (min, max)	0.9 (0.4, 2.4)	0.9 (0.5, 1.6)	1.0 (0.6, 1.6)	1.3 (0.5, 2.6)	1.8 (0.8, 4.8)	1.5 (0.6, 1.6)	1.3 (1.3, 1.7)	1.2 (0.6, 2.6)	1.2 (0.8, 1.6)	0.9 (0.7, 1.0)	3.8 (3.8, 3.8)	1.7 (1.7, 1.7)	1.1 (0.4, 4.8)
Missing	0 (0%)	1 (4.2%)	0 (0%)	0 (0%)	0 (0%)	0 (0%)	0 (0%)	0 (0%)	0 (0%)	0 (0%)	0 (0%)	0 (0%)	1 (0.8%)
**Mesocolon length (widest point, cm)**													
Mean (SD)	26.1 (9.4)	22.4 (5.2)	23.2 (7.7)	30.3 (11.9)	22.7 (9.0)	29.3 (11.4)	44.0 (24.4)	28.4 (7.7)	18.8 (1.1)	38.0 (28.3)	38.0 (NA)	48.0 (NA)	26.3 (10.8)
Median (min, max)	26.0 (8.3, 45.0)	21.0 (14.0, 39.0)	21.0 (14.0, 41.0)	27.3 (9.5, 62.0)	20.5 (11.3, 46.0)	27.0 (18.0, 46.0)	47.5 (11.0, 70.0)	29.5 (18.0, 36.6)	18.8 (18.0, 19.5)	38.0 (18.0, 58.0)	38.0 (38.0, 38.0)	48.0 (48.0, 48.0)	24.0 (8.3, 70.0)
**Mesocolon volumn (approx, cm^3^)**													
Mean (SD)	799.0 (570.7)	713.7 (643.8)	834.2 (803.3)	1187.5 (657.1)	753.6 (732.5)	2527.6 (2692.7)	1524.9 (1189.8)	1053.7 (631.1)	775.7 (206.0)	1364.6 (1548.0)	3553.0 (NA)	673.9 (NA)	974.3 (935.0)
Median (min, max)	691.9 (2.4, 2288.0)	514.5 (84.5, 2886.0)	728.5 (81.0, 3936.0)	1228.1 (96.9, 2480.0)	499.5 (68.0, 2369.9)	1633.5 (307.5, 6720.0)	1694.0 (132.0, 2579.5)	1034.2 (361.2, 1785.0)	775.7 (630.0, 921.4)	1364.6 (270.0, 2459.2)	3553.0 (3553.0, 3553.0)	673.9 (673.9, 673.9)	699.7 (2.4, 6720.0)
**Time (first pass, min)**													
Mean (SD)	59.3 (20.3)	43.0 (19.0)	42.7 (28.6)	45.1 (21.6)	36.3 (14.3)	40.0 (12.8)	33.3 (23.2)	37.5 (16.3)	43.0 (4.2)	58.0 (45.3)	115.0 (NA)	35.0 (NA)	46.6 (22.9)
Median (min, max)	59.0 (22.0, 105.0)	37.5 (18.0, 80.0)	36.5 (24.0, 158.0)	45.0 (15.0, 110.0)	33.5 (20.0, 75.0)	38.0 (25.0, 60.0)	31.0 (11.0, 60.0)	40.0 (17.0, 53.0)	43.0 (40.0, 46.0)	58.0 (26.0, 90.0)	115.0 (115.0, 115.0)	35.0 (35.0, 35.0)	42.0 (11.0, 158.0)
**Time (total, min)**													
Mean (SD)	63.6 (30.8)	44.7 (21.4)	44.5 (28.6)	45.1 (21.6)	36.6 (14.2)	47.4 (19.4)	35.3 (20.9)	37.8 (16.6)	43.0 (4.2)	58.0 (45.3)	295.0 (NA)	72.0 (NA)	50.4 (34.0)
Median (min, max)	59.0 (22.0, 180.0)	37.5 (18.0, 90.0)	37.0 (24.0, 158.0)	45.0 (15.0, 110.0)	33.5 (20.0, 75.0)	40.0 (25.0, 74.0)	32.0 (17.0, 60.0)	40.5 (17.0, 53.0)	43.0 (40.0, 46.0)	58.0 (26.0, 90.0)	295.0 (295.0, 295.0)	72.0 (72.0, 72.0)	44.5 (15.0, 295.0)
**TAT (days)**													
Mean (SD)	4.9 (3.8)	4.8 (3.8)	4.1 (2.2)	4.0 (2.2)	4.3 (2.5)	4.0 (1.0)	5.3 (2.8)	4.8 (1.7)	5.0 (0.0)	4.0 (2.8)	9.0 (NA)	4.0 (NA)	4.5 (3.0)
Median (min, max)	4.0 (1.0, 22.0)	4.5 (1.0, 20.0)	3.5 (2.0, 10.0)	3.5 (2.0, 9.0)	4.0 (2.0, 9.0)	4.0 (3.0, 5.0)	5.5 (2.0, 8.0)	4.5 (3.0, 7.0)	5.0 (5.0, 5.0)	4.0 (2.0, 6.0)	9.0 (9.0, 9.0)	4.0 (4.0, 4.0)	4.0 (1.0, 22.0)
**TAT - business days (days)**													
Mean (SD)	3.6 (2.7)	3.5 (2.6)	3.1 (1.2)	3.0 (1.5)	3.0 (1.9)	3.2 (0.4)	3.8 (2.1)	3.3 (1.3)	3.0 (0.0)	3.0 (1.4)	6.0 (NA)	3.0 (NA)	3.3 (2.0)
Median (min, max)	3.0 (1.0, 16.0)	3.0 (1.0, 14.0)	3.0 (2.0, 6.0)	2.0 (2.0, 7.0)	2.0 (1.0, 7.0)	3.0 (3.0, 4.0)	3.5 (2.0, 6.0)	3.0 (2.0, 5.0)	3.0 (3.0, 3.0)	3.0 (2.0, 4.0)	6.0 (6.0, 6.0)	3.0 (3.0, 3.0)	3.0 (1.0, 16.0)
**Examiner credentials**													
PA	18 (62.1%)	16 (66.7%)	12 (60.0%)	15 (83.3%)	8 (66.7%)	1 (20.0%)	2 (50.0%)	3 (75.0%)	2 (100%)	1 (50.0%)	0 (0%)	0 (0%)	78 (63.9%)
Resident	1 (3.4%)	0 (0%)	0 (0%)	0 (0%)	2 (16.7%)	0 (0%)	0 (0%)	0 (0%)	0 (0%)	0 (0%)	0 (0%)	0 (0%)	3 (2.5%)
Fellow	0 (0%)	1 (4.2%)	0 (0%)	0 (0%)	0 (0%)	0 (0%)	0 (0%)	0 (0%)	0 (0%)	0 (0%)	0 (0%)	0 (0%)	1 (0.8%)
Missing	10 (34.5%)	7 (29.2%)	8 (40.0%)	3 (16.7%)	2 (16.7%)	4 (80.0%)	2 (50.0%)	1 (25.0%)	0 (0%)	1 (50.0%)	1 (100%)	1 (100%)	40 (32.8%)
**Examiner experience**													
<1 year	2 (6.9%)	3 (12.5%)	3 (15.0%)	0 (0%)	3 (25.0%)	0 (0%)	0 (0%)	0 (0%)	0 (0%)	0 (0%)	0 (0%)	0 (0%)	11 (9.0%)
1–5 years	10 (34.5%)	5 (20.8%)	4 (20.0%)	5 (27.8%)	6 (50.0%)	0 (0%)	1 (25.0%)	3 (75.0%)	2 (100%)	0 (0%)	0 (0%)	0 (0%)	36 (29.5%)
6 or more	7 (24.1%)	9 (37.5%)	5 (25.0%)	10 (55.6%)	1 (8.3%)	1 (20.0%)	1 (25.0%)	0 (0%)	0 (0%)	1 (50.0%)	0 (0%)	0 (0%)	35 (28.7%)
Missing	10 (34.5%)	7 (29.2%)	8 (40.0%)	3 (16.7%)	2 (16.7%)	4 (80.0%)	2 (50.0%)	1 (25.0%)	0 (0%)	1 (50.0%)	1 (100%)	1 (100%)	40 (32.8%)
**Neoadjuvant treatment**													
No	6 (20.7%)	19 (79.2%)	8 (40.0%)	17 (94.4%)	10 (83.3%)	5 (100%)	2 (50.0%)	4 (100%)	2 (100%)	2 (100%)	1 (100%)	1 (100%)	77 (63.1%)
Unknown	0 (0%)	0 (0%)	0 (0%)	0 (0%)	1 (8.3%)	0 (0%)	1 (25.0%)	0 (0%)	0 (0%)	0 (0%)	0 (0%)	0 (0%)	2 (1.6%)
Yes	23 (79.3%)	5 (20.8%)	12 (60.0%)	1 (5.6%)	1 (8.3%)	0 (0%)	1 (25.0%)	0 (0%)	0 (0%)	0 (0%)	0 (0%)	0 (0%)	43 (35.2%)
pT0	7 (24.1%)	1 (4.2%)	2 (10.0%)	2 (11.1%)	1 (8.3%)	0 (0%)	0 (0%)	0 (0%)	0 (0%)	0 (0%)	0 (0%)	0 (0%)	13 (10.7%)
pTis	0 (0%)	0 (0%)	0 (0%)	1 (5.6%)	0 (0%)	0 (0%)	0 (0%)	0 (0%)	0 (0%)	0 (0%)	0 (0%)	0 (0%)	1 (0.8%)
pT1	2 (6.9%)	3 (12.5%)	1 (5.0%)	1 (5.6%)	1 (8.3%)	0 (0%)	0 (0%)	0 (0%)	0 (0%)	0 (0%)	0 (0%)	0 (0%)	8 (6.6%)
pT2	7 (24.1%)	3 (12.5%)	6 (30.0%)	2 (11.1%)	3 (25.0%)	2 (40.0%)	0 (0%)	2 (50.0%)	0 (0%)	1 (50.0%)	0 (0%)	0 (0%)	26 (21.3%)
pT3	11 (37.9%)	13 (54.2%)	8 (40.0%)	8 (44.4%)	3 (25.0%)	2 (40.0%)	3 (75.0%)	1 (25.0%)	0 (0%)	1 (50.0%)	1 (100%)	0 (0%)	51 (41.8%)
pT4	0 (0%)	0 (0%)	0 (0%)	0 (0%)	0 (0%)	0 (0%)	0 (0%)	1 (25.0%)	0 (0%)	0 (0%)	0 (0%)	0 (0%)	1 (0.8%)
pT4a	0 (0%)	1 (4.2%)	1 (5.0%)	3 (16.7%)	2 (16.7%)	1 (20.0%)	1 (25.0%)	0 (0%)	0 (0%)	0 (0%)	0 (0%)	0 (0%)	9 (7.4%)
pT4b	0 (0%)	2 (8.3%)	0 (0%)	0 (0%)	1 (8.3%)	0 (0%)	0 (0%)	0 (0%)	1 (50.0%)	0 (0%)	0 (0%)	0 (0%)	4 (3.3%)
Missing	2 (6.9%)	1 (4.2%)	2 (10.0%)	1 (5.6%)	1 (8.3%)	0 (0%)	0 (0%)	0 (0%)	1 (50.0%)	0 (0%)	0 (0%)	1 (100%)	9 (7.4%)
**Lesion category**													
Dysplasia/other	2 (6.9%)	0 (0%)	0 (0%)	1 (5.6%)	0 (0%)	0 (0%)	0 (0%)	0 (0%)	1 (50.0%)	0 (0%)	0 (0%)	0 (0%)	4 (3.3%)
No residual tumor	6 (20.7%)	3 (12.5%)	5 (25.0%)	2 (11.1%)	1 (8.3%)	0 (0%)	0 (0%)	0 (0%)	0 (0%)	0 (0%)	0 (0%)	0 (0%)	17 (13.9%)
Tumor	21 (72.4%)	21 (87.5%)	15 (75.0%)	14 (77.8%)	10 (83.3%)	5 (100%)	4 (100%)	4 (100%)	1 (50.0%)	2 (100%)	1 (100%)	1 (100%)	99 (81.1%)
Intramucosal cancer with high grade dysplasia	0 (0%)	0 (0%)	0 (0%)	1 (5.6%)	0 (0%)	0 (0%)	0 (0%)	0 (0%)	0 (0%)	0 (0%)	0 (0%)	0 (0%)	1 (0.8%)
Adenoma with dysplasia	0 (0%)	0 (0%)	0 (0%)	0 (0%)	1 (8.3%)	0 (0%)	0 (0%)	0 (0%)	0 (0%)	0 (0%)	0 (0%)	0 (0%)	1 (0.8%)

### 3.2 Volume of mesenteric tissue

The total volume of mesenteric tissue analyzed is shown in Table 1. The rectum and transverse colon had the least total volume of mesenteric tissue at 673.9 cm^3^, while the sigmoid colon had the most volume of mesenteric tissue at 17,128.9 cm^3^. When examining the median volume of the colon samples, the “descending colon and sigmoid colon” had the largest mesenteric volume at 3553.0 cm^3^, while the “cecum” had the smallest mesenteric volume at 499.5 cm^3^. The Kruskal-Wallis rank sum test indicated that none of the groups are statistically significantly different from all groups, and pairwise comparisons using the Wilcoxon rank sum test demonstrated that there were no statistically significant differences found between any two groups.

### 3.3 Time to search LNs

**FIGURE 1:**
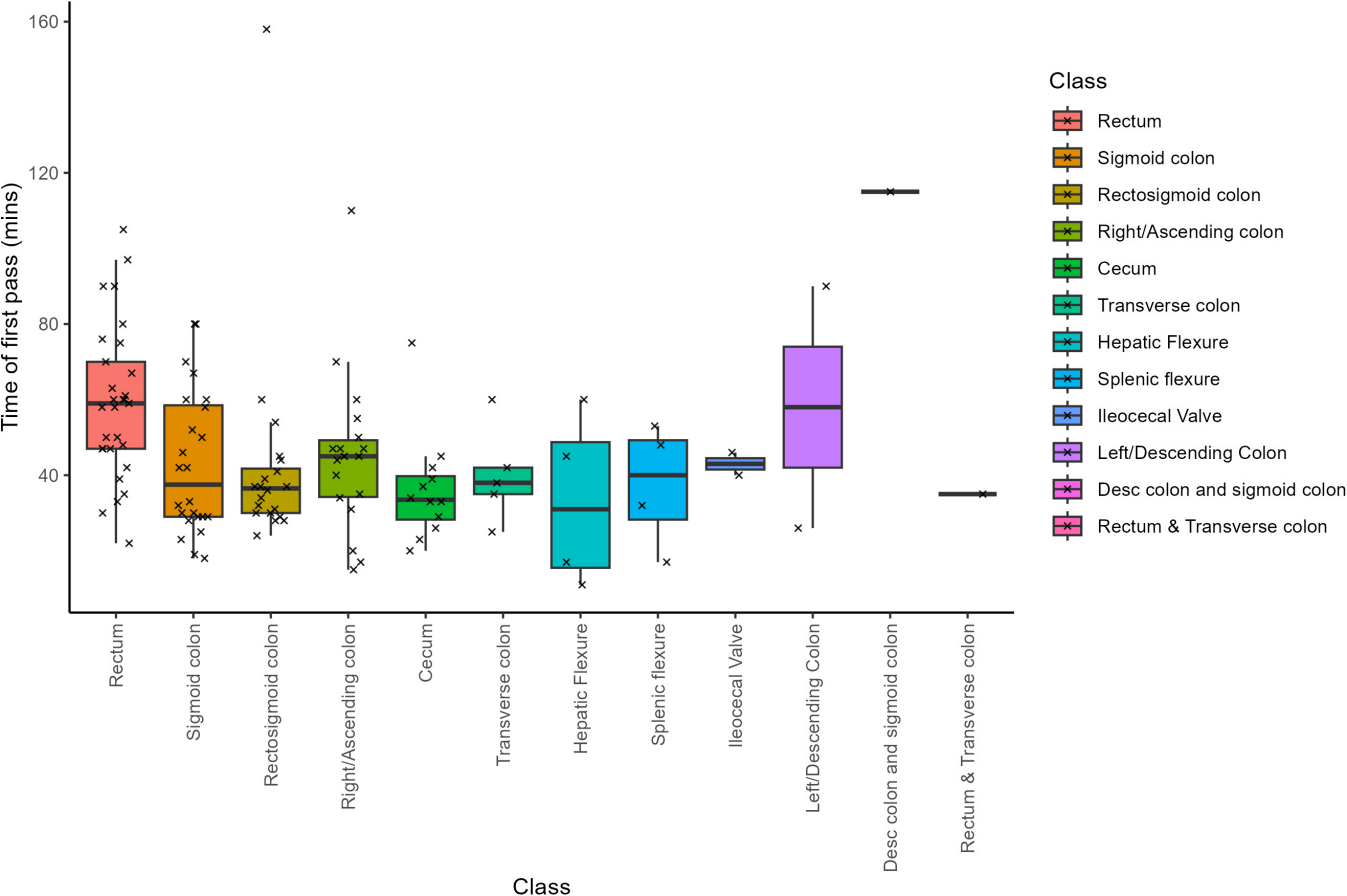
Lymph node search time by cancer location.

The mean time to search for LNs was 50.4 min (SD = 34.0) across all specimens, with individual search times ranging from 15 to 295 min (Figure 1, Supplementary Figure 1). When broken down by tissue type, the average search time varied considerably–from 35.25 minutes for hepatic flexure samples to 295 min for descending colon and sigmoid colon samples. Variability in search times also differed by tissue type; for instance, samples from the ileocecal valve had a low standard deviation of 4.24 min, whereas those from the left/descending colon exhibited a high standard deviation of 45.24 min.

The Kruskal-Wallis test confirmed that there were statistically significant differences in search times among tissue types (Chi-squared = 22.005, df = 7, *p*-value = 0.003; Supplementary Table 1). Pairwise comparisons using the Wilcoxon rank sum test (with Benjamin-Hochberg adjustments) revealed that the rectum required significantly different search times compared to several other groups. Specifically, the rectum differed significantly from the sigmoid colon (*p* = 0.047), rectosigmoid colon (*p* = 0.007), and cecum (*p* = 0.008). Other pairwise comparisons between tissue types did not reach statistical significance.

When accounting for total processing time–which includes additional time spent beyond the initial LN search–the significant differences were maintained for rectum versus rectosigmoid colon and rectum versus cecum, but the difference between rectum and sigmoid colon was no longer statistically significant (Supplementary Table 2). In 14 out of 122 cases, extra search time was required; this additional time was particularly notable in samples from the descending and sigmoid colons, where an extra 150 min was allocated on average (Supplementary Figure 1). Samples from the rectum and transverse colon also required more time beyond the initial search, ranking just behind the descending and sigmoid colon groups.

Overall, these results indicate that LN search times vary substantially by tissue type, with both rectal samples and those from the descending and sigmoid colon requiring significantly longer processing times. This variability likely reflects the anatomical challenges inherent in these regions and emphasizes the importance of tailored approaches during LN retrieval.

### 3.4 Average count of LNs at the macro and micro-count level

**FIGURE 2:**
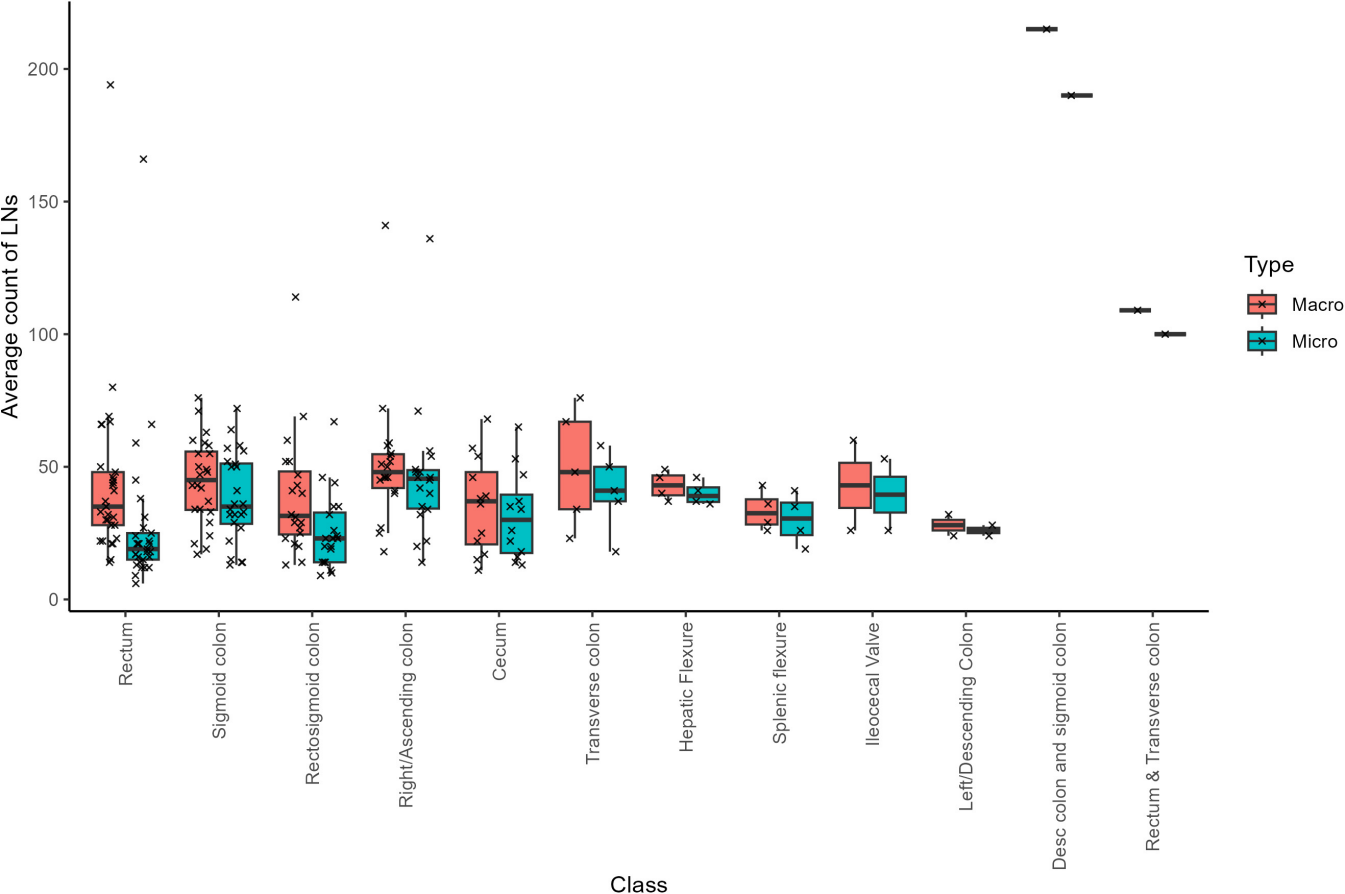
Total average “macro” and “micro” LN counts based on cancer location.

Across all specimens, the dissector recorded an average of 45.1 LNs per sample at the macroscopic level (SD = 29.1; Supplementary Figure 2). Subsequent microscopic evaluation confirmed an average of 35.7 LNs per sample (SD = 26.8). The macroscopic LN counts had a median of 41, ranging from a minimum of 11 to a maximum of 215 LNs, while the microscopic counts showed a median of 32, with values ranging from 6 to 190 LNs. Notably, the specimen from the descending colon and sigmoid colon region exhibited the highest LN counts–215 at the macroscopic level and 190 at the microscopic level (Figure 2). In contrast, the lowest mean LN counts differed by evaluation method: the “left/descending colon” samples had the lowest mean macroscopic count (28 LNs, SD = 5.7), whereas the “rectosigmoid colon” samples had the lowest mean microscopic count (25.5 LNs, SD = 14.4).

### 3.5 LN counts and the presence and/or absence of neoadjuvant chemotherapy

We investigated whether neoadjuvant chemotherapy affected LN counts at both the macroscopic and microscopic levels. At the macroscopic level (Supplementary Figure 2A), a Wilcoxon Rank Sum test indicated no statistically significant difference in LN counts between patients who received neoadjuvant chemotherapy and those who did not (*W* = 1952, *p* = 0.105). In contrast, at the microscopic level (Supplementary Figure 2B), the LN counts differed significantly between the two groups (*W* = 2423, *p* = 2.676 e−05). These findings suggest that while neoadjuvant chemotherapy does not substantially alter the initial (macroscopic) LN count, it is associated with a significant reduction in the number of LNs confirmed upon microscopic evaluation.

### 3.6 Comparing macro and micro LN counts with cancer locations

We next examined whether cancer locations correlated with LN counts. First, we assessed whether any tissue type exhibited a gross LN count that differed significantly from others. After removing low-count classes, the Kruskal-Wallis test indicated that there were no statistically significant differences in macroscopic LN counts among tissue types (chi-squared = 9.2786, df = 7, *p* = 0.233).

A similar assessment was performed for microscopic LN counts. In this case, the Kruskal-Wallis test revealed a statistically significant difference among tissue groups (chi-squared = 25.524, df = 7, *p* = 0.001). Pairwise comparisons using the Wilcoxon rank sum test (with Benjamin-Hochberg adjustments) identified significant differences between several tissue pairs: rectum versus sigmoid colon (*p* = 0.019), rectum versus right/ascending colon (*p* = 0.009), and rectosigmoid colon versus right/ascending colon (*p* = 0.017) (Supplementary Table 3).

To capture the heterogeneity inherent in routinely processed specimens, our methodology deliberately included outliers. These outliers are crucial for a comprehensive evaluation of data variability, which we quantified using the median and Median Absolute Deviation (MAD) (Supplementary Table 4). Notably, specimens from the Rectum exhibited the most substantial variability (highest MAD) at both macroscopic and microscopic levels, while those from the hepatic flexure showed the lowest variability. Furthermore, the highest median LN counts were observed in the right/ascending colon, whereas the lowest median counts were seen in the rectosigmoid colon at the macroscopic level and in the rectum at the microscopic level. Additionally, the data spread (as measured by MAD) was highest in the Transverse colon for macroscopic counts and in the sigmoid colon for microscopic counts, with hepatic flexure specimens consistently displaying the lowest MAD scores.

Finally, a comparison of LN counts between macroscopic and microscopic examinations across tissue locations (Figure 2) revealed that rectum samples exhibited the most significant discrepancy, while splenic flexure samples showed the minimal difference between the two assessment methods. These findings highlight the nuanced variability in LN assessment across anatomical locations, offering valuable insights into the pathological evaluation of lymphatic spread in colorectal carcinomas. Furthermore, this discrepancy is not unexpected, as gross examination relies on palpation and visual cues, which can misidentify vessels or fibrous tissue as LNs, while small or soft nodes may be missed entirely. Microscopic review remains the definitive standard for confirming lymph node identity.

### 3.7 Correlating macro and/or micro-LN counts with various factors

To elucidate potential correlations between specimen characteristics and LN counts, we conducted a series of analyses focusing on mesocolon tissue dimensions, T-stage, and neoadjuvant status.

#### 3.7.1 Mesocolon tissue dimensions

Supplementary Figure 3 illustrates that there is no robust correlation between the dimensions of the mesocolon and LN counts at either the macroscopic or microscopic levels. The data show considerable variability. Some specimens with large mesocolon dimensions exhibit only average LN counts, while some with small to medium-sized tissues display substantially high LN counts. This indicates that an increase in mesocolon volume does not necessarily lead to a higher number of detected LNs, suggesting that other factors may play more influential roles.

#### 3.7.2 T-stage and LN counts

Motivated by previous studies linking tumor enlargement to increased LN metastasis prevalence (36), we investigated the relationship between T-stage and LN counts. As shown in Supplementary Figure 4 and detailed in Supplementary Table 5, T-stage has a statistically significant effect on the total number of LNs confirmed microscopically (ANOVA: *F* = 2.55, *p* = 0.018). In contrast, as illustrated in Supplementary Figure 5 and Supplementary Table 6, T-stage does not significantly impact the number of cancer-positive LNs (*F* = 1.613, *p* = 0.140). These results suggest that while higher T-stages are associated with an overall increase in LN retrieval, they do not necessarily correspond with a higher number of cancer-positive nodes.

#### 3.7.3 Multivariable analysis of LN counts

Beyond univariate comparisons, we performed multivariable ANOVAs to determine which factors significantly influence LN counts.

For macroscopic LN counts (Supplementary Table 7), the analysis identified sample class, T-stage, and total sample time as statistically significant predictors. In particular, an extended search time correlates with a higher macroscopic LN yield–a relationship that remains robust despite potential outlier effects. Mesocolon tissue volume, tumor presence, and examiner experience (singleExaminer_years) were not significant predictors.

For microscopic LN counts (Supplementary Table 8), significant factors included sample class, neoadjuvant status, total sample time, T-stage, and the total macroscopic LN count. Notably, the macroscopic count emerged as a potent predictor of the microscopic count, reinforcing the intuitive expectation that a higher number of LNs identified during gross examination will lead to a higher confirmation rate microscopically. Again, mesocolon tissue volume did not show a significant effect.

Overall, these analyses highlight that while mesocolon tissue dimensions do not predict LN counts, factors such as T-stage, total search time, and the initial macroscopic LN count are critical determinants in both macroscopic and microscopic LN assessments.

### 3.8 Cancer-positive LNs and correlations with total LN counts; cancer location, neoadjuvant status, and tumor characteristics

**FIGURE 3:**
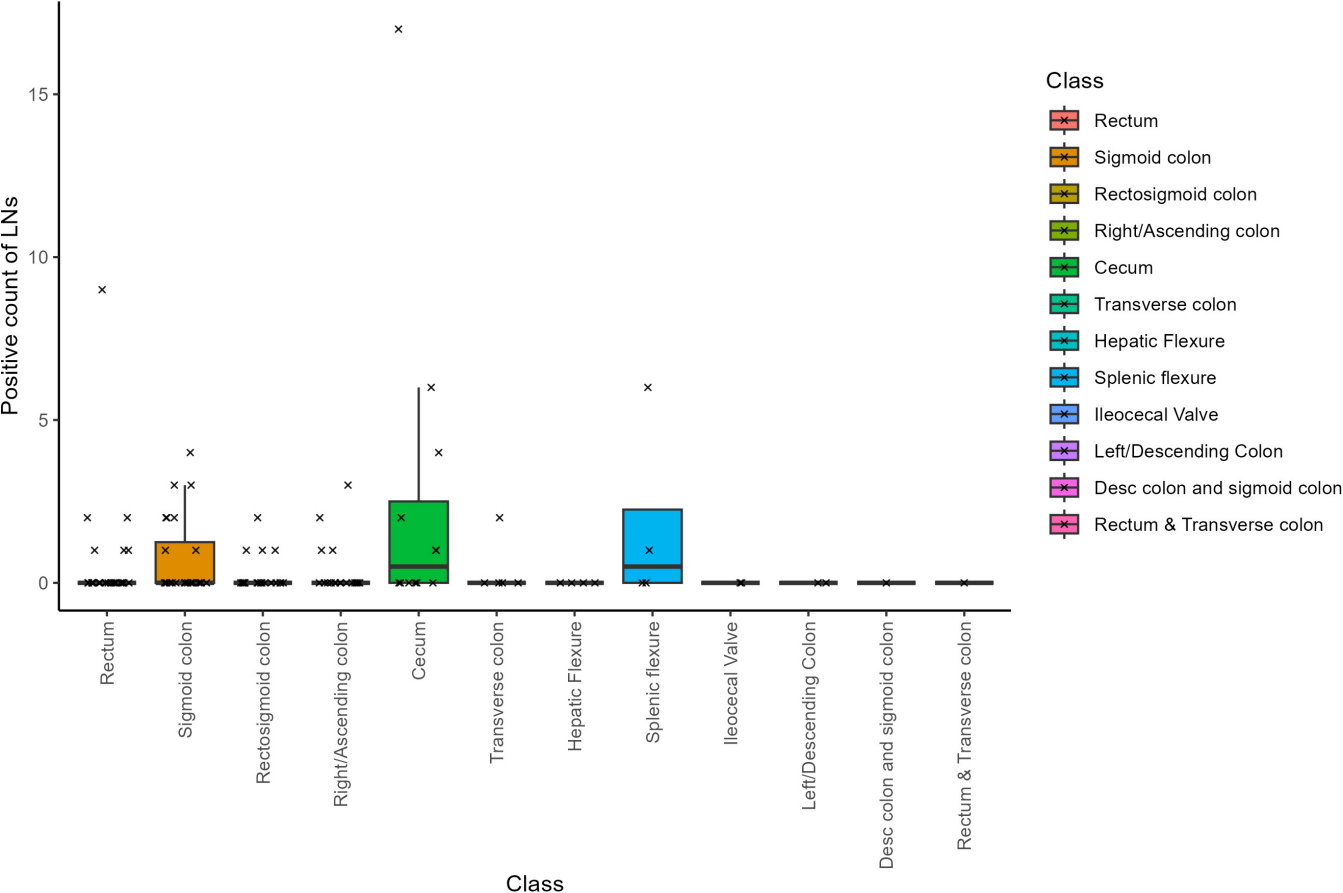
Examining positive lymph node counts and their correlation with tumor specimen location.

Across the 122 cases, a total of 4354 LNs were submitted and confirmed under the microscope, of which 4268 were negative and 86 were malignant–indicating that approximately 2% of the LNs were cancer positive. On average, there were 0.705 positive LNs per case. Notably, samples from the cecum exhibited the highest incidence of cancer-positive LNs, with a mean of 2.6 (SD = 4.9; median = 0.5; Table 1). In contrast, no cancer-positive LNs were detected in five sample types: Hepatic Flexure, ileocecal valve, left/descending colon, descending colon and sigmoid colon, and rectum and transverse colon (Figure 3). This absence likely reflects the limited sample sizes for these categories, and a larger dataset might reveal a different distribution.

This finding may reflect either limitations in sample size or the influence of confounding biological factors, such as tumor biology or variability in metastatic spread (2). Neoadjuvant therapy initially appeared to influence positive LN counts; however, two cases with unknown neoadjuvant status–one of which was a clear outlier–introduced considerable variability in the data distribution (Supplementary Figure 6). Rather than exclude this data point, we chose to retain it to reflect the real-world variability inherent in routine colorectal cancer pathology. We re-assessed the corresponding macroscopic and microscopic pathology reports and confirmed the documented LN counts. Given the nature of the study and scope constraints, no additional histologic or paraffin block review was conducted. Nonetheless, we acknowledge that the presence of such outliers can affect overall group-level comparisons, and future studies with larger, stratified cohorts may be better equipped to explore these rare but influential observations.

Overall, our search for significant predictors of total positive LN counts yielded no robust findings. As detailed in Supplementary Table 9, the multivariable model exhibited relatively high residual variance, suggesting that the factors included (sample class, neoadjuvant status, total sample time, T-stage, and total LN counts) did not fully explain the variability in positive LN counts. Intriguingly, sample class emerged as a somewhat more influential variable than neoadjuvant therapy; however, neither reached strong significance. A Wilcoxon non-parametric test indicated a marginal effect of neoadjuvant therapy on positive LN counts (*p* = 0.036), though the effect size appears minimal. Furthermore, there was no significant correlation between positive LN counts and mesocolon volume (Supplementary Figure 7). These observations emphasize the complex interplay of factors influencing the detection of cancer-positive LNs, with no single variable strongly predicting positive LN counts within our dataset.

### 3.9 Min/mean/max size of collected LNs, and correlations with sample class and neoadjuvant status

Across the 122 samples, the variable for the smallest LN size showed only five distinct values, indicating limited variability. Notably, one sample was an outlier with a smallest LN size of 0.9 cm, whereas the vast majority of samples had smallest LN sizes between 0.1 and 0.3 cm (Supplementary Figure 8). Overall, LN sizes ranged from 0.1 to 4.8 cm. The median size of the smallest LN per sample was 0.2 cm, while the median size of the largest LN per sample was 1.1 cm, representing the typical range encountered in our analysis.

The size of the largest recovered LN varied significantly across sample classes (Supplementary Figure 9 and Supplementary Table 10). Specifically, when comparing between classes with at least 4 samples, significant differences in largest LN size were observed between: cecum and rectum samples (*p* = 0.028), cecum and sigmoid colon samples (*p* = 0.028), cecum and rectosigmoid colon samples (*p* = 0.028), right/ascending colon and rectum (*p* = 0.031), right/ascending colon and sigmoid colon (*p* = 0.031), right/ascending colon and rectosigmoid colon (*p* = 0.031), and hepatic flexure and rectosigmoid colon.

Additionally, LNs tended to be smaller in cases where the patient received neoadjuvant chemotherapy (Supplementary Figure 10; *p* = 1.147 e−04). These findings suggest that both the anatomical origin of the specimen and neoadjuvant treatment status can influence the size of the largest LN recovered.

### 3.10 Macroscopic LN counts versus microscopic confirmation and mean absolute percentage error (MAPE)

The MAPE is a robust metric that quantifies the discrepancy between predicted values and their actual (ground truth) values. In our study, we used MAPE to assess the difference between the LN counts obtained during macroscopic dissection and those confirmed by microscopic examination. MAPE is calculated by averaging the absolute percentage errors for all data points–each determined by taking the absolute difference between the macroscopic and microscopic LN counts, divided by the microscopic count. A lower MAPE indicates a closer agreement between estimated and actual LN counts.

**FIGURE 4:**
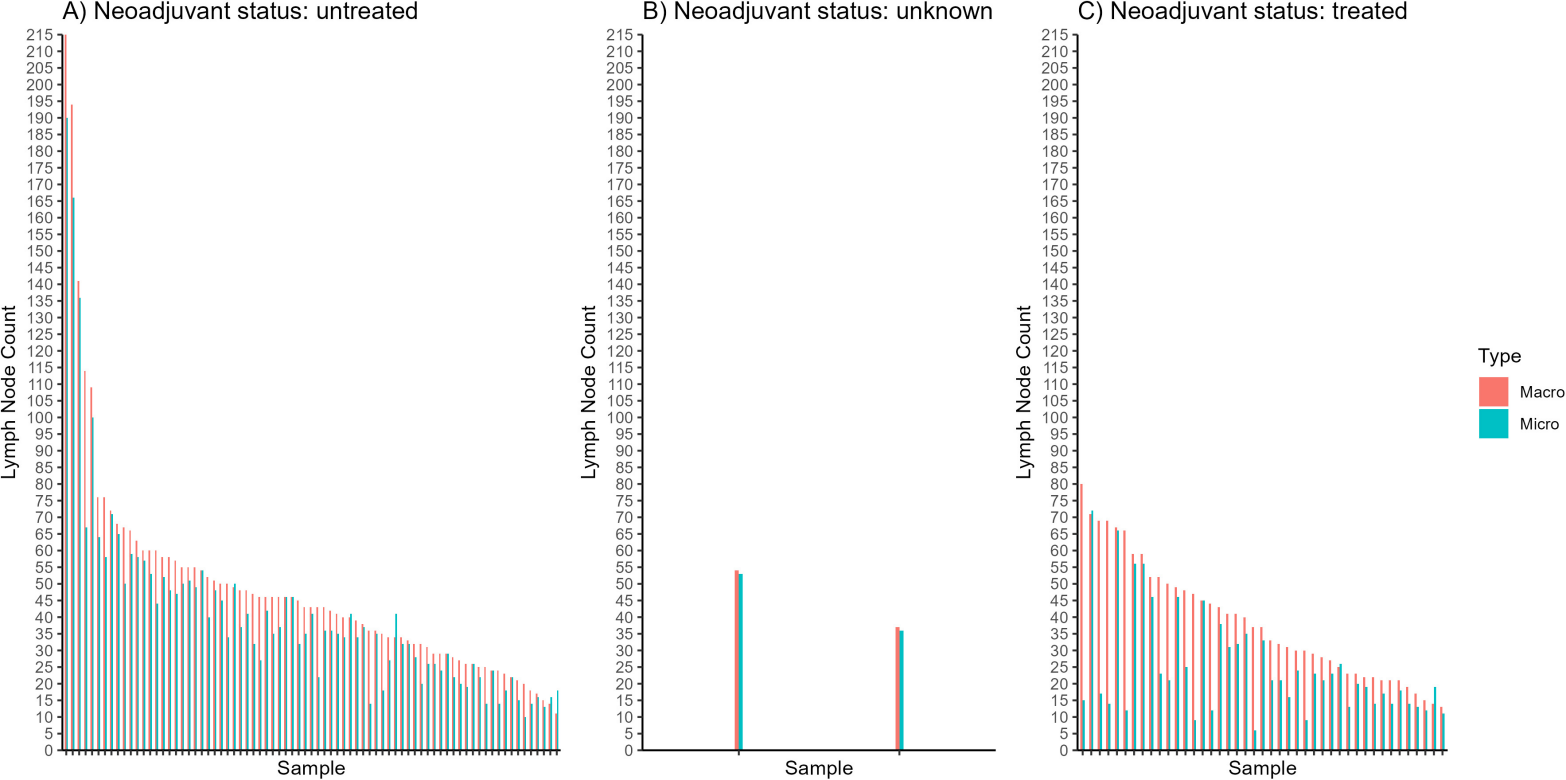
Graphical comparisons between macroscopic (“macro”) and microscopic (“micro”) lymph node counts for individual samples of patients who **(A)** did not receive neoadjuvant chemotherapy, **(B)** had an “unknown” status of neoadjuvant chemotherapy, and **(C)** received neoadjuvant chemotherapy.

Among the 122 cases analyzed, 106 cases exhibited higher macroscopic LN counts than those confirmed microscopically, with an average overestimation of 11.08 LNs per case (Figure 4). Conversely, 8 cases showed lower macroscopic counts (underestimations) by an average of 3.125 LNs, while 8 cases demonstrated identical counts between the two methods. Overall, the average MAPE across all cases was 50.18%. Notably, cases treated with neoadjuvant therapy had a substantially higher MAPE of 97.87%, compared to 25.37% for untreated cases.

To contextualize these percentages, we multiplied the MAPE values by the corresponding average microscopic LN counts for each group. For neoadjuvant-treated cases, the average microscopic LN count was 25.07, so a MAPE of 97.87% translates to an average discrepancy of approximately 0.9787 × 25.07 ≈ 24.5 LNs per case. In contrast, for untreated cases, where the average microscopic LN count was 41.39, a MAPE of 25.37% corresponds to an average error of about 0.2537 × 41.39 ≈ 10.5 LNs per case. This analysis highlights the substantial variability–and potential for error–in manual LN identification techniques, particularly in the context of neoadjuvant therapy.

### 3.11 Counts versus grosser experience

Among the 122 cases, examiner details were available for 82 observations. The professional breakdown was as follows: 78 PAs, 3 residents, and 1 fellow. The majority of gross dissections in our study were performed by PAs, which reflects common practice within the pathology workflow in the United States. Our intent was not to evaluate or compare international practice models or justify the value of PAs, but rather to characterize LN retrieval outcomes as they occur under typical U.S. protocols.

To examine how error metrics vary with examiner experience, we evaluated the MAPE for different experience groups (Supplementary Table 11). Although the mean MAPE showed notable differences among the groups, the median MAPE values were relatively consistent. To further explore this, we plotted the Absolute Percentage Error (APE) against examiner experience (Supplementary Figure 11). The resulting boxplots revealed no substantial differences in error magnitude–apart from a few extreme outliers. Statistical testing confirmed that the differences in APE among the experience groups were not significant (*p* = 0.491), suggesting that examiner experience did not have a discernible impact on the error magnitude in our dataset.

In addition, we compared the LN counts obtained via macroscopic and microscopic examinations across the different levels of examiner experience (Supplementary Table 12). Examiners with less than 1 year of experience reported an average macroscopic LN count of 35 and an average microscopic LN count of 17.83. Examiners with 1–5 years of experience observed an average macroscopic LN count of 38.55 and an average microscopic LN count of 15.85, while those with six or more years of experience identified an average macroscopic LN count of 44.31 and an average microscopic LN count of 22.39. However, the Kruskal-Wallis rank sum test indicated that the differences in macroscopic LN counts (*p* = 0.303) and microscopic LN counts (*p* = 0.075) across these experience levels were not statistically significant.

These findings highlight the complexity and challenges inherent in accurate LN counting during pathological examinations, and suggest that differences in LN counts are not solely attributable to examiner proficiency.

### 3.12 Macroscopic versus microscopic LN count errors by cancer location and region-based classification

In line with our previous analyses, we first evaluated the discrepancies between macroscopic and microscopic LN counts across different cancer locations (i.e., sample classes). As shown in Supplementary Figure 12, the boxplots reveal considerable variability in error magnitudes among classes, with some classes exhibiting pronounced outliers. A filtered dataset (excluding classes with low counts) was then analyzed using the Kruskal-Wallis rank sum test, and the resulting pairwise comparisons (Supplementary Table 13) revealed several significant differences between cancer locations. These findings suggest that the challenge of accurate LN identification may vary by anatomical site.

Interestingly, our data also indicate that increased examiner experience does not uniformly translate into greater accuracy across all cancer locations. This observation deviates from the initial expectation that higher experience would consistently reduce errors, particularly in more challenging specimens. Instead, it appears that certain cancer locations pose inherent difficulties that are not fully mitigated by examiner proficiency. Although we were unable to control simultaneously for examiner experience and cancer location due to dataset limitations, these preliminary insights highlight the need for future research to explore the complex interplay between examiner proficiency, cancer location, and LN count accuracy.

To complement the “sample class” approach, we additionally categorized specimens into broader anatomical “regions” using operator notes, specimen measurements, and surgical intent: Left (L), Right (R), Transverse (T), Left–Transverse (L,T), Right–Transverse (R,T), and Right–Transverse–Left (R,T,L). This distribution of cancer locations across regions is shown in Supplementary Figure 13, illustrating that while location and region often overlap (e.g., many “right/ascending colon” specimens fall under R), they do not align perfectly. When we compared macroscopic LN counts among these regions (Supplementary Figure 14), the R,T,L group typically exhibited the highest total LN counts, likely reflecting more extensive multi-segment resections. In contrast, L and R samples largely overlapped, although R showed a modest shift toward higher LN yields.

Although the Kruskal-Wallis test for microscopic LN counts indicated a potential overall difference among regions (*p* = 0.014; Supplementary Table 16), subsequent pairwise comparisons revealed no statistically significant differences between any specific region pairs. Neither macroscopic LN counts (*p* = 0.190; Supplementary Table 17) nor cancer-positive LN counts (*p* = 0.317; Supplementary Table 18) differed significantly across regions. These results suggest that, within our cohort, the broader anatomical region approach was not a strong predictor of LN yields or positivity.

A similar pattern emerged in microscopic LN counts (Supplementary Figure 15), showing a somewhat larger gap between L and R than was observed macroscopically. However, these differences in total LN yield did not translate into variations in cancer-positive LN counts, as Supplementary Figure 16 demonstrated no statistically significant disparities in the number of positive nodes among the six regions.

Overall, these analyses suggest that both the conventional “sample class” system and the broader “region” classification capture variability in LN yields, yet neither classification alone fully explains the observed differences in LN positivity or retrieval accuracy. Integrating these classification schemes with additional factors–such as examiner experience, specimen complexity, or tumor morphology–and a larger sample size could offer a more nuanced perspective on the challenges of LN retrieval in colorectal specimens.

### 3.13 Time spent on LN search versus error rates

To investigate the influence of various variables on the error rates observed in macroscopic LN counts, we constructed an ANOVA model using the APE between macroscopic and microscopic LN counts as the dependent variable. This analysis was performed on the “singleGrosser” dataset, which includes examiner experience as one of the independent variables. The model incorporated cancer class, neoadjuvant status, total sample time, T-stage, mesocolon tissue volume, and examiner experience (singleExaminer_years).

The ANOVA results (Supplementary Table 14) show that the residual variability (Sum Sq = 25.405) substantially exceeds the variability explained by the independent variables (combined Sum Sq ≈ 12.26), indicating that much of the variation in error rates remains unaccounted for by the factors we measured. Importantly, cancer class (*p* = 0.045) emerged as a significant predictor of APE. In contrast, total sample time, neoadjuvant status, T-stage, mesocolon tissue volume, and examiner experience did not have statistically significant effects. These findings suggest that the intrinsic challenges associated with specific cancer locations–and the additional time required to search them–may drive higher error rates, rather than examiner experience alone.

This is particularly evident in the near bimodal distribution of APE versus search time (Supplementary Figure 17), where most samples showed low error rates, yet a distinct subset (all from rectal examinations) exhibited both long search times and high error rates. Thus, spending more time on LN searches appears to be associated with increased errors in certain cases, reflecting inherent difficulties in processing complex specimens rather than simply a matter of inexperience. In practice, this indicates that additional strategies–such as enhanced training, refined protocols, or supplemental technologies–may be necessary to improve accuracy in challenging cases. Furthermore, the substantial unexplained variability suggests that other, unmeasured factors likely contribute to LN count errors and warrant further investigation.

### 3.14 Impact of sample volume on LN counts and tissue examination rate

We revisited the time versus volume plots to discern any overarching trends between the volume of mesocolon tissue and the time dedicated to its examination. Although larger samples tended to require slightly longer examination times, the correlation was weak (R^2^ = 0.111), suggesting that tissue volume does not consistently predict examination time (Supplementary Figure 18). When stratified by sample class (Supplementary Figure 19), the relationship varied across classes; notably, samples classified as “rectosigmoid colons” and “descending colon and sigmoid colon” exhibited a positive correlation between total examination time and tissue volume, while other classes showed less pronounced trends.

Next, we calculated the tissue examination rate–defined as the volume of tissue examined per minute (accounting for all time spent, including multiple passes)–across the entire cohort. The analysis yielded a mean rate of 21.94 cm^3^/min, a median of 15.13 cm^3^/min, and a standard deviation of 22.62 cm^3^/min, with rates ranging from 0.04 to 176.84 cm^3^/min (Supplementary Table 15). This wide range highlights substantial variability in examination efficiency among samples.

By investigating tissue examination rates by sample class (Supplementary Figure 20), we observed that the transverse colon displayed the largest spread between minimum and maximum rates. However, this extreme variability appears to be driven primarily by a single outlier. Thus, while there may be differences in efficiency across sample classes, caution is needed when interpreting these differences, as they might reflect occasional anomalies rather than systematic trends.

We also compared examination rates for samples processed by multiple examiners versus a single examiner (Supplementary Figure 21). Although there was an observable trend toward a lower examination rate in cases involving multiple examiners, the difference was not statistically significant – potentially reflecting the additional time and effort required for examiners to coordinate their work. This finding is consistent with our measurement approach, where total time is recorded in “people-minutes.” In this framework, while having more examiners could split the workload, any efficiency gains appear to be offset by the added “coordination overhead” – the time and effort needed for examiners to communicate, stay aligned on procedures, and consolidate findings – yielding examination rates that are quite similar.

Finally, we explored the relationship between tissue examination rate and macroscopic LN counts (Supplementary Figures 22, 23). Our analysis revealed a slight inverse correlation: higher tissue examination rates (indicative of faster, possibly less meticulous dissections) were associated with lower macroscopic LN counts. This suggests that a slower, more careful dissection approach may enhance LN detection, particularly in more challenging specimens.

Overall, these findings indicate that while sample volume has only a modest impact on examination time, the efficiency of tissue examination varies considerably and appears to be influenced by both inherent tissue characteristics and the dissection approach. Importantly, the data suggest that slower, more deliberate examination may lead to improved LN retrieval, highlighting a potential trade-off between speed and accuracy in pathological assessments.

### 3.15 Average turnaround time for path report

The average turnaround time (TAT) for the pathology report was 3.34 business days, with most samples being reported within 2–3 business days (Supplementary Figure 24). Only two observations exceeded 1.5 times the interquartile range: one case took 14 business days and another 16 business days. No significant differences in TAT were observed based on cancer location (Supplementary Figure 25), nor was there any change in TAT for samples that required additional passes during the examination process (Supplementary Figure 26).

## 4 Discussion

Accurate LN staging is critical for managing colorectal cancer (CRC), given its strong prognostic value and role in guiding adjuvant chemotherapy (37). Although current guidelines recommend retrieving at least 12 LNs to ensure accurate staging (17), recent studies suggest that examining higher LN counts may be associated with improved outcomes (17, 23, 24, 38) – potentially because a stronger host immune response leads to the development or preservation of more detectable lymph nodes, rather than being solely a reflection of more thorough pathological assessment. However, the interplay between immune response and LN detectability, along with other factors such as dissection techniques, LN size, and tumor location, remains understudied–particularly in prospective settings. To help address this gap, our study was designed with prospective data collection during real-time gross dissections in routine clinical workflows. This forward-looking approach involved standardized documentation at the time of dissection and direct recording of variables such as LN counts, dissection times, and mesocolon dimensions, providing operational insights that are not typically captured in retrospective analyses.

In our study, the average dissection time for colorectal specimens was about 50 min, aligning with previous reports that note a range of 30–50 min (39, 40). Although overall times were similar across cancer locations, rectal specimens often exhibited significantly longer durations. This may reflect both anatomical complexity–such as a narrow pelvic cavity and dense mesorectal fat–and procedural challenges posed by neoadjuvant therapy, which is frequently administered for rectal cancer and is known to induce fibrosis and obscure nodal architecture (41–45). Additionally, specimens from the descending and sigmoid colon occasionally necessitated additional passes. Notably, one outlier case involved a large-volume, non-neoadjuvant-treated descending/sigmoid colon specimen with over 215 macroscopic LNs identified across three passes, requiring a total dissection time of 295 min. Across the entire cohort, approximately 95% of dissections were completed within 90 min, suggesting that prolonged dissection durations were uncommon under the specific workflows and resource settings at the Mayo Clinic. When extended processing times did occur, they were typically associated with high lymph node yields or specimens with greater anatomical complexity. However, our dataset does not allow definitive conclusions about whether these extended times were driven by specimen-related factors, institutional workflow constraints, or individual operator experience. While larger specimens occasionally trended toward longer dissection durations, the modest correlation observed suggests that sample volume alone does not fully account for variability–highlighting the multifactorial nature of grossing effort. Further study, including multi-center analyses, may help disentangle these contributors to better understand dissection variability.

We identified an average of 45.1 potential LNs during gross examination (macroscopic counts) and 35.7 LNs upon microscopic confirmation. Neoadjuvant chemotherapy did not affect the gross potential LN counts, but it was associated with significantly lower microscopic counts, likely due to treatment effects on lymphatic tissue (41, 46). These results are consistent with previous studies demonstrating reduced LN counts in neoadjuvant-treated rectal cancers compared to surgery alone (41, 47). Additionally, we observed a positive correlation between T-stage and microscopic LN counts, suggesting that more advanced tumors–possibly due to higher immunogenicity or more aggressive surgical intervention–yield more LNs (28, 48, 49). However, T-stage did not predict the number of cancer-positive nodes, indicating that advanced disease does not necessarily equate to more metastatic involvement.

The greatest discrepancy between macroscopic and microscopic counts occurred in rectal specimens, where non-lymphatic tissues were sometimes misidentified as LNs. In contrast, specimens from the splenic flexure showed minimal differences. Interestingly, in 8 cases, the microscopic count exceeded the gross count, suggesting that some LNs were missed during the initial dissection, likely because the LNs were too small to be detected grossly. In such cases, representative sections of mesenteric tissue, particularly when submitted during low-yield dissections, may have incidentally captured non-palpable LNs, leading to higher microscopic counts than gross/macro counts. While future studies might explore the feasibility of submitting all remaining mesenteric tissue for histologic examination, even comprehensive submission may not guarantee detection. Small LNs buried deep in fatty tissue may still be overlooked unless tissue processing, paraffin embedding, and sectioning protocols are optimized – challenges that must also be weighed against significant cost constraints (15, 42, 50).

Lymph node size also played a role in identification; the smallest grossly suspected LN measured 0.1 cm and the largest 4.8 cm. Prior research indicates that 83% of initially missed LNs are 2 mm or smaller (50) and that many metastatic LNs are found in nodes with diameters ≤ 5 mm, while nodes larger than 10 mm are less frequently metastatic (51). This underscores the need for meticulous dissection to avoid missing small, yet clinically significant, LNs.

For the first time in the literature, we report notable error rates when comparing gross LN counts to microscopic confirmation, with an overall MAPE of 50.18% and a striking 97.87% among neoadjuvant-treated cases. Rather than representing an anomaly, these elevated error rates likely reflect real-world challenges in LN identification, particularly under biologically complex conditions such as neoadjuvant therapy, which can induce LN regression, fibrosis, and scarring that obscure nodal architecture and reduce gross palpability (41–45). These effects, coupled with the inherent subjectivity of gross dissection (where LNs may be overcalled based on palpation but not confirmed microscopically) introduce further variability and help explain the discrepancy between macroscopic and microscopic counts. Although no prior studies have directly quantified LN search error rates in this way, related work has shown that re-sampling can alter staging (15, 50), reinforcing the need for consistency and accuracy in LN retrieval. By introducing MAPE as a novel metric, our study provides a quantitative benchmark for assessing LN retrieval discrepancies across protocols, operators, or institutions. Ultimately, these findings highlight the limitations of manual dissection and the opportunity for improved protocols or supportive technologies to enhance LN retrieval–particularly in treatment-altered tissues.

The average turnaround time (TAT) for pathology reports was 3.3 business days, well within the College of American Pathologists’ 4-days recommendation (17). TATs were not significantly influenced by cancer location or the need for additional LN searches, likely reflecting efficient workflows at the Mayo Clinic. In our cohort, two outlier cases exceeded the 4-days TAT guideline, potentially due to specimen complexity or the need for additional histologic workup–factors previously associated with extended turnaround times in large or challenging specimens (52). Nonetheless, TAT variability due to such factors across institutions warrants further investigation, as delays in pathology reports can impact patient care coordination (52–54).

Our data did not reveal significant differences in LN retrieval based on the experience level of the personnel performing gross examination. In fact, several studies indicate that less experienced pathologists or PAs may retrieve more LNs (31, 55) –possibly due to greater diligence and available time. This finding highlights that the anatomical complexity of specimens may have a greater influence on LN counts than operator experience, a hypothesis that requires further study. It is also important to contextualize these findings within the typical pathology workflow in the U.S. In our study, gross dissections were predominantly performed by PAs trained under standardized protocols at the Mayo Clinic. This reflects common practice in many North American institutions, where PAs routinely perform initial specimen handling before microscopic review by a pathologist. While this model generally supports procedural consistency, we acknowledge that LN retrieval outcomes may vary internationally depending on personnel, training backgrounds, and institutional practices. These differences should be considered when interpreting the generalizability of our findings across different healthcare systems. Furthermore, although our analysis did not identify statistically significant differences in error rates between personnel with varying experience levels, this may reflect the standardized protocols and shared tools used during dissection. Future studies should investigate technique-specific or inter-rater variability among dissectors using a controlled study design.

Economically, our findings underscore the potential cost implications of LN retrieval errors, particularly in neoadjuvant cases. Errors can lead to increased expenses due to additional histology processing, slide preparation, and pathologist review. Histology processing costs range from approximately $25 to >$50 per block, with special stains or immunohistochemistry adding $10–$50 per slide (56, 57). Reprocessing of tissues due to missed LNs can increase workloads by as much as 700% (58), and reviewing erroneous slides by a pathologist can cost $83 to >$166 per hour (56). These additional costs not only strain laboratory resources but also risk impacting patient staging if cancer-positive LNs are missed. Märkl et al. estimate that under-staging occurs in 2%–5% of cases (51), while Tran et al. reported that 8.5% of patients had changes in high-risk features after LN re-sampling (15) –findings that have significant clinical and financial implications, especially in the context of adjuvant chemotherapy decisions for stage II colon cancer without high-risk features (59). Future research should include dedicated cost-effectiveness studies to evaluate how the frequency of reprocessing events and the incremental costs of extended pathology review translate into broader financial and clinical impacts. Although our sample size was sufficient for observational analysis, certain subgroup comparisons (e.g., outlier dissections) were limited by small category sizes. Larger, multi-institutional cohorts will be essential to better assess how sample complexity and LN retrieval errors influence pathology turnaround time and resource utilization.

A limitation of our study is the relatively small sample size, which restricts the generalizability of our conclusions. Additionally, while we observed significant differences in microscopic LN counts across cancer locations, this study was not designed to evaluate the underlying biological reasons–such as regional differences in lymphatic density, stromal composition, or vascular architecture–that may contribute to these variations. Future studies should incorporate larger datasets and systematic methodologies to investigate these potential anatomical and histopathologic factors. Similarly, while we did not assess the spatial relationship between LNs and the tumor, future investigations using 3D reconstruction or annotated tissue maps could determine whether proximity to the primary tumor influences nodal involvement or detection. A more mechanistic understanding of why discrepancies occur–particularly in neoadjuvant-treated and anatomically complex cases–could also be pursued through dedicated histological or molecular studies focused on lymphatic regression, tissue fibrosis, or immune remodeling. This could inform refined grossing strategies or risk-adapted staging protocols.

In conclusion, accurate LN retrieval is essential for CRC staging and treatment planning. While our study reveals challenges in the LN search process–especially in neoadjuvant-treated and rectal specimens–it also opens the door for improvements in dissection techniques and resource allocation. Enhancing LN retrieval accuracy will be critical to optimizing patient outcomes while managing the increasing demands on pathology laboratories.

## Data Availability

The original contributions presented in this study are included in this article/Supplementary material, further inquiries can be directed to the corresponding author.
