# Supplementary material for: A prospective analysis of lymph node retrieval in colorectal cancer: discrepancies, neoadjuvant impact, and practical implications
Source: Front Med (Lausanne). 2025 Oct 1;12:1611170. doi: 10.3389/fmed.2025.1611170 (PMC12521114; doi:10.3389/fmed.2025.1611170)
Supplement: Supplementary file 1 [file Data_Sheet_1.pdf]

## Supplementary Material

# 1 Supplementary Figures

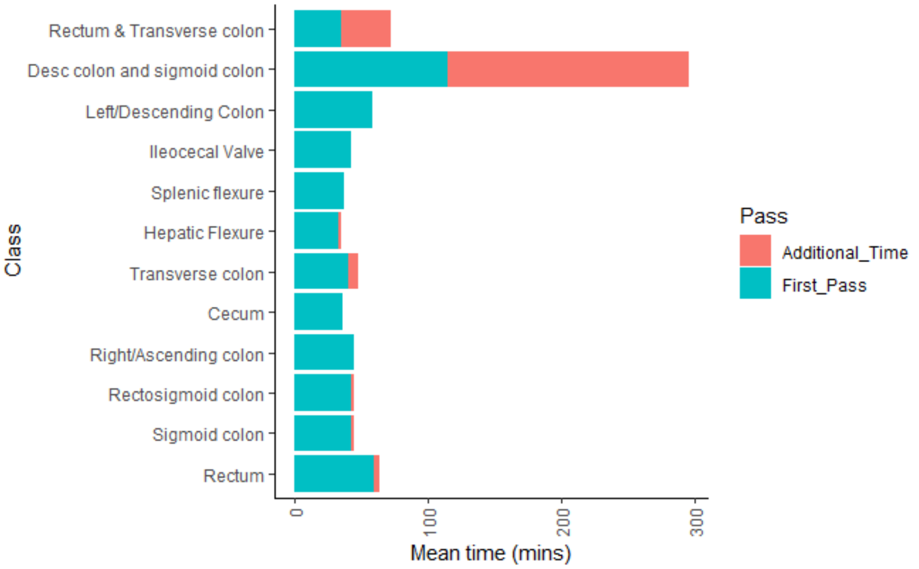


**Supplementary Figure 1.** Mean search time for first pass and after additional passes for lymph node searches by tumor specimen location.

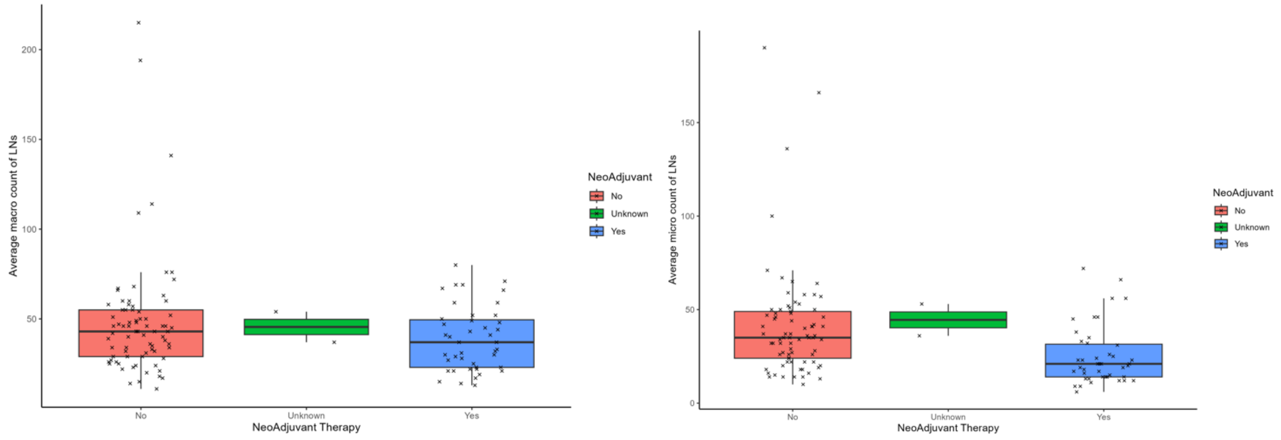


**Supplementary Figure 2.** Total average count of LNs per sample at the 'macroscopic' versus 'micro' level. A) Macro LN counts for samples with or without neoadjuvant chemotherapy. B) Micro LN counts for samples with or without neoadjuvant chemotherapy.

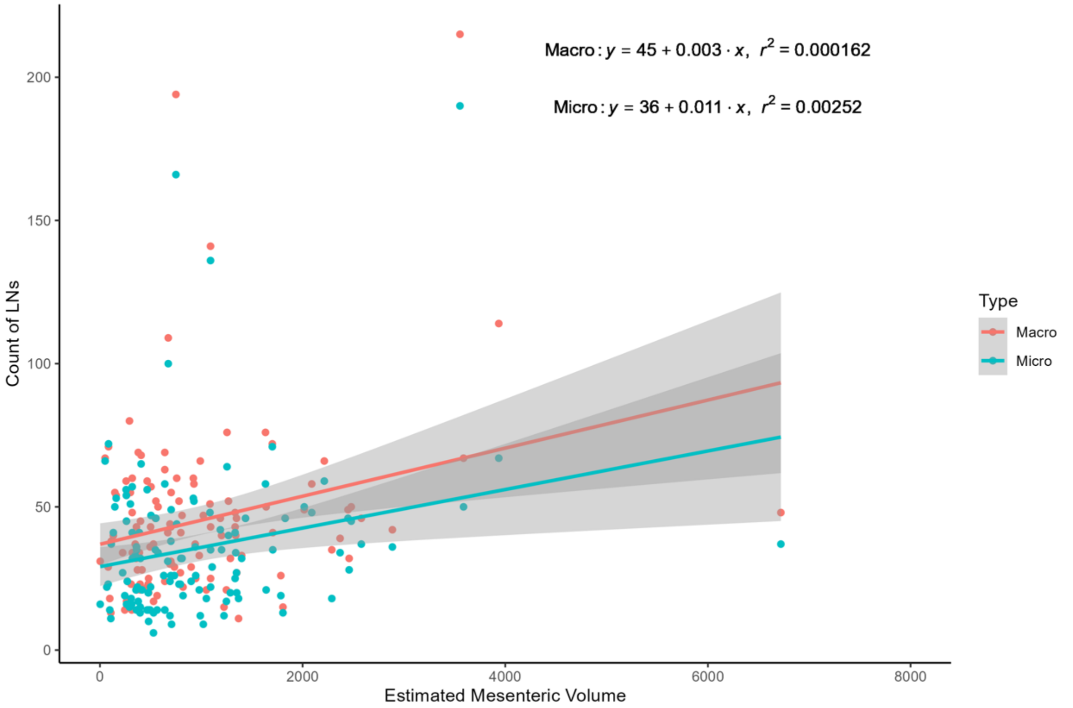


**Supplementary Figure 3.** Total lymph node counts (both macro and micro LNs) correlated with estimated mesenteric tissue volume.

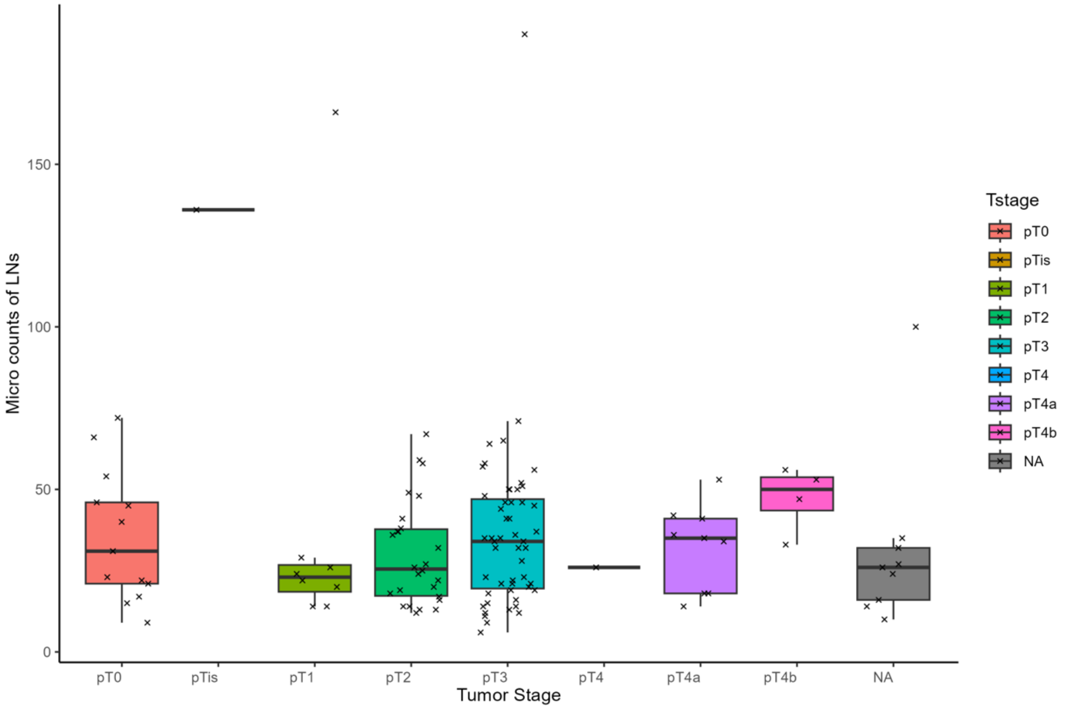


**Supplementary Figure 4.** Total micro lymph node counts correlated with T-stage.

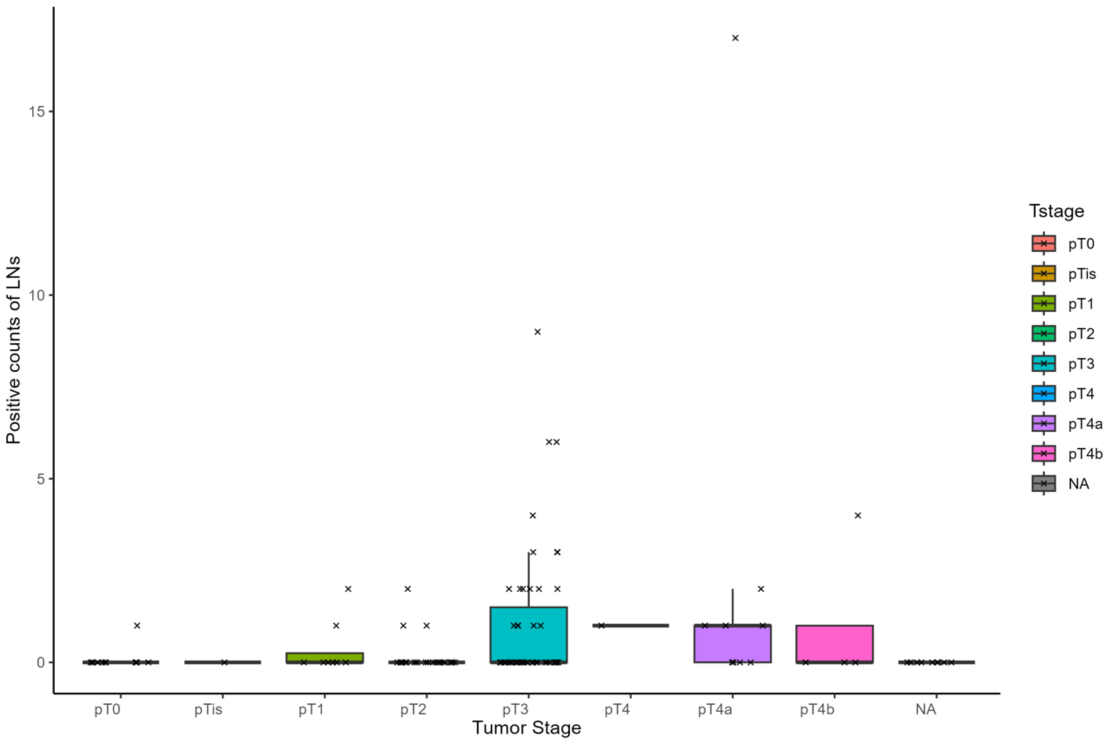


**Supplementary Figure 5.** Total cancer-positive lymph node counts confirmed under the microscope and its relationship with T stage.

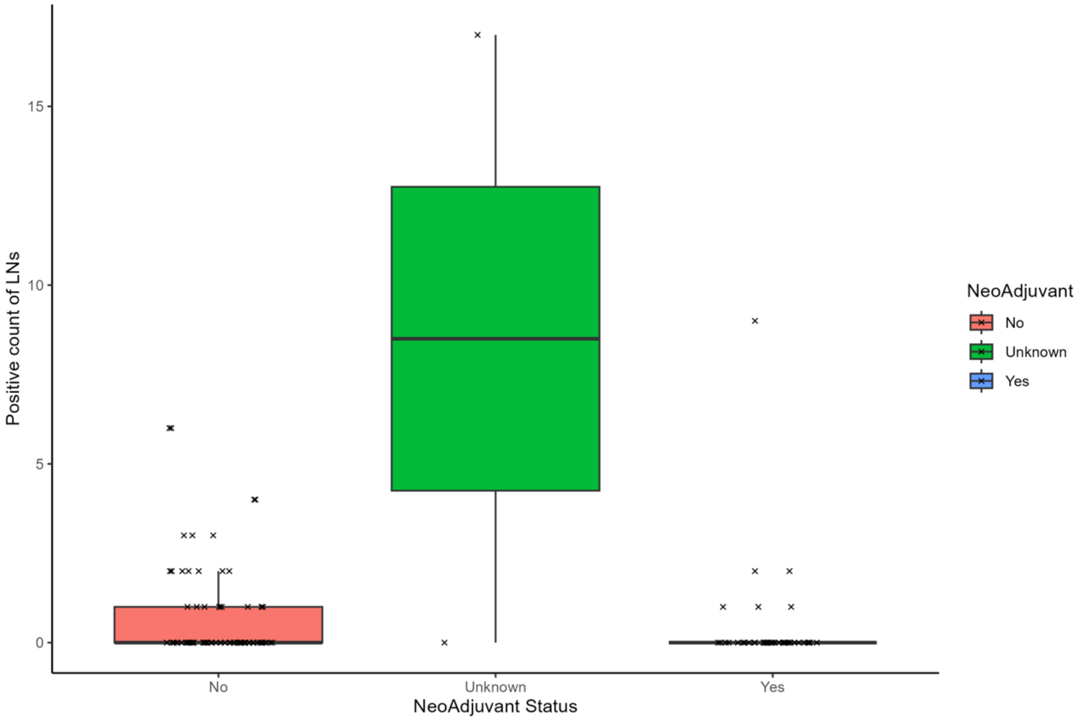


**Supplementary Figure 6.** Positive LN counts (at microscopic level) and their correlation with neoadjuvant status.

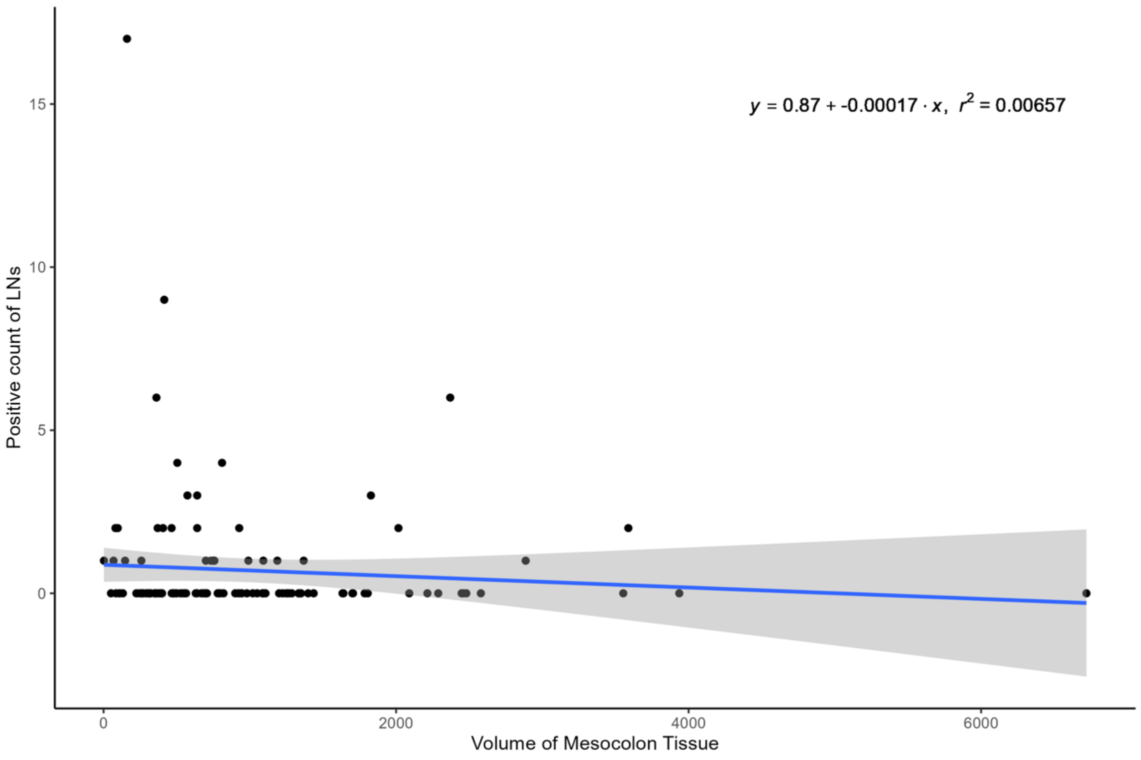


**Supplementary Figure 7.** Positive LN counts and their correlation with mesocolon volume.

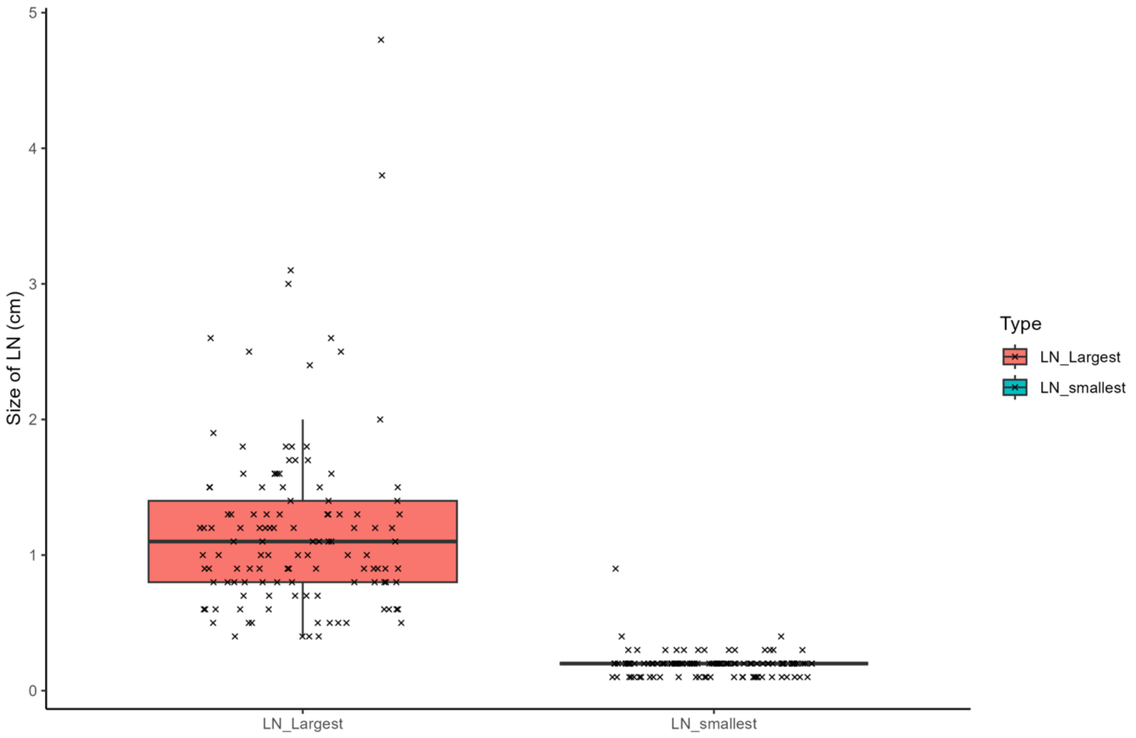


**Supplementary Figure 8.** Comparing the largest and smallest lymph node sizes (in cm).

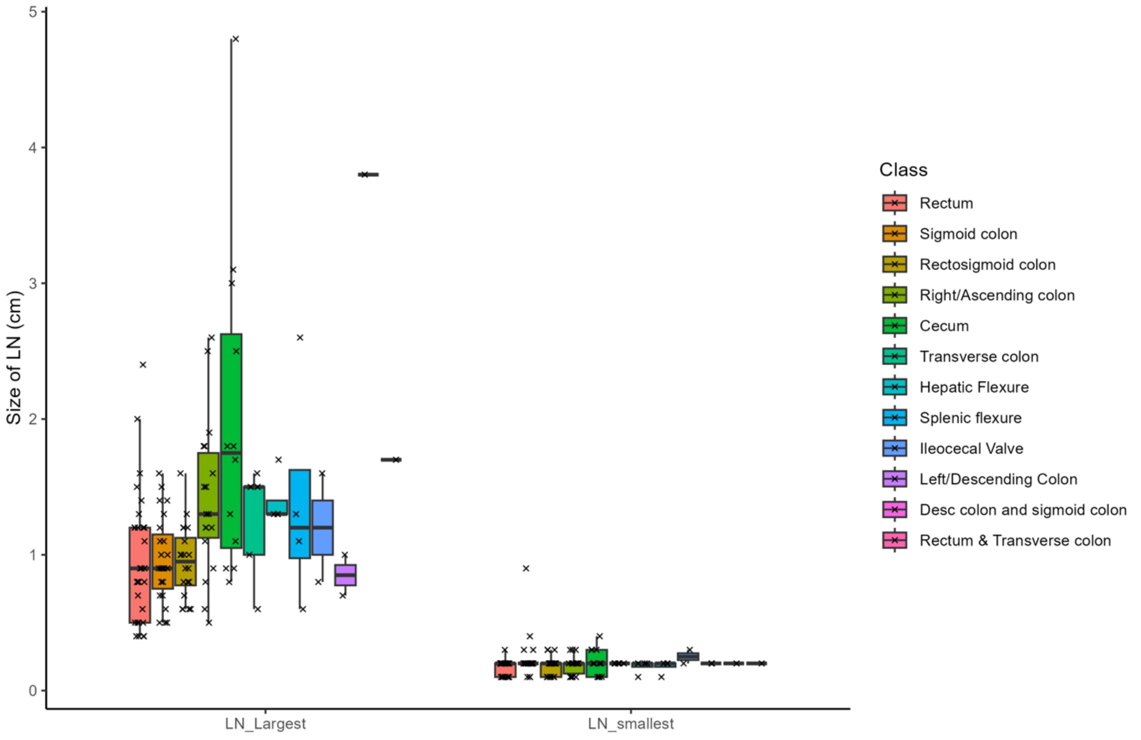


**Supplementary Figure 9.** Comparing the largest and smallest lymph node sizes (in cm) by tumor specimen location.

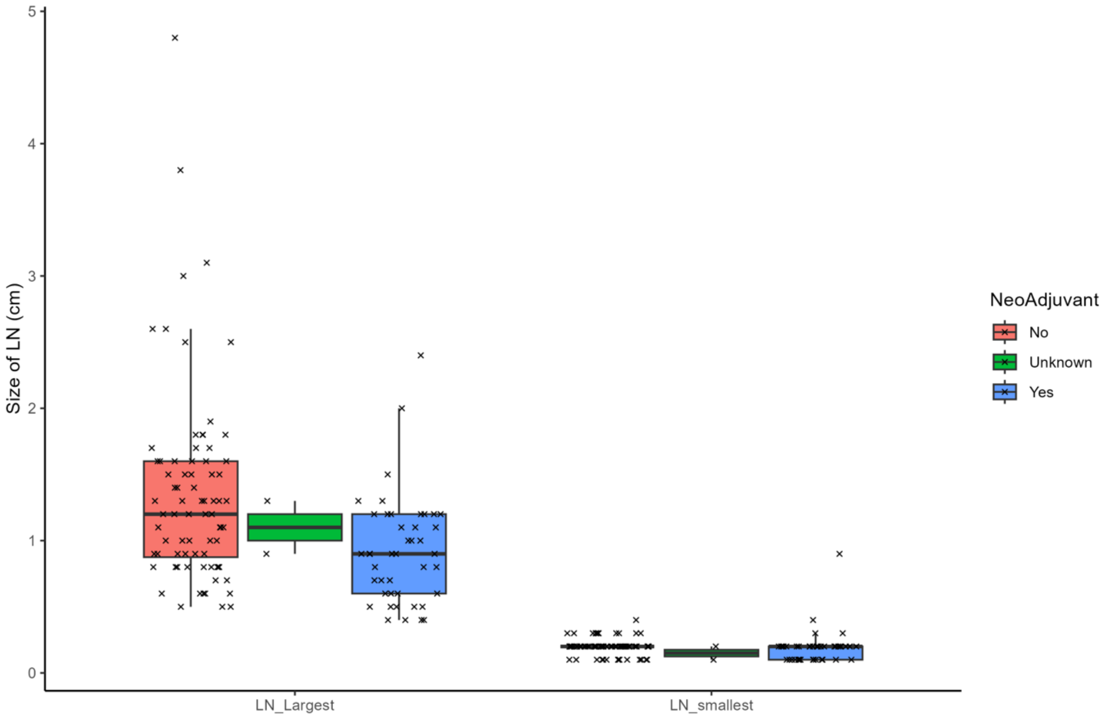


**Supplementary Figure 10.** Comparing the largest and smallest lymph node sizes (in cm) by neoadjuvant treatment status.

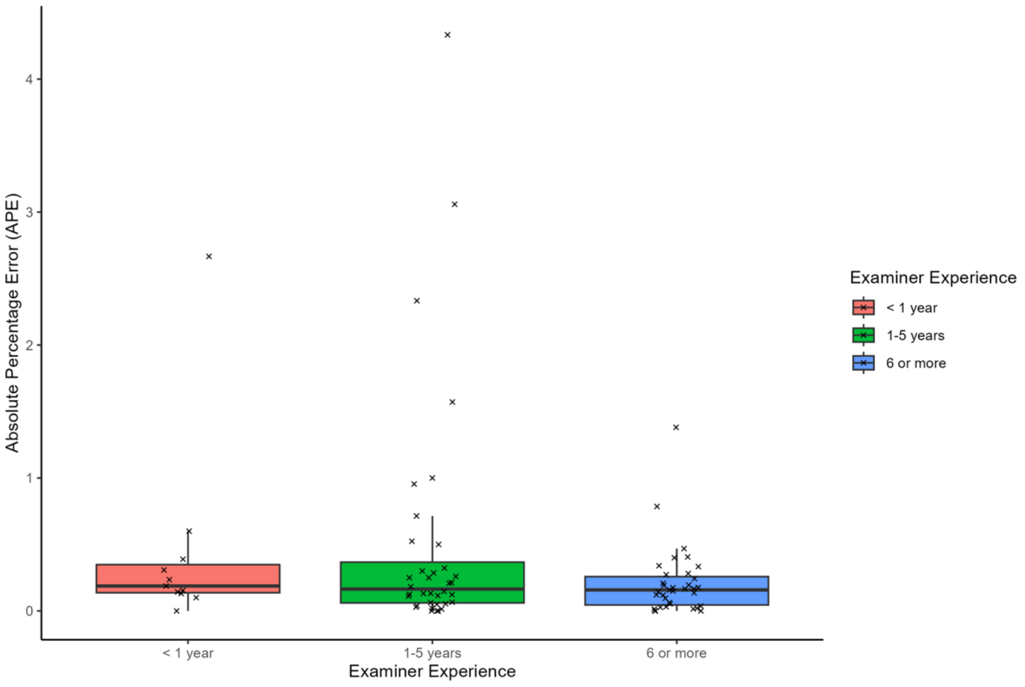


**Supplementary Figure 11.** Examining absolute percentage error by examiner experience.

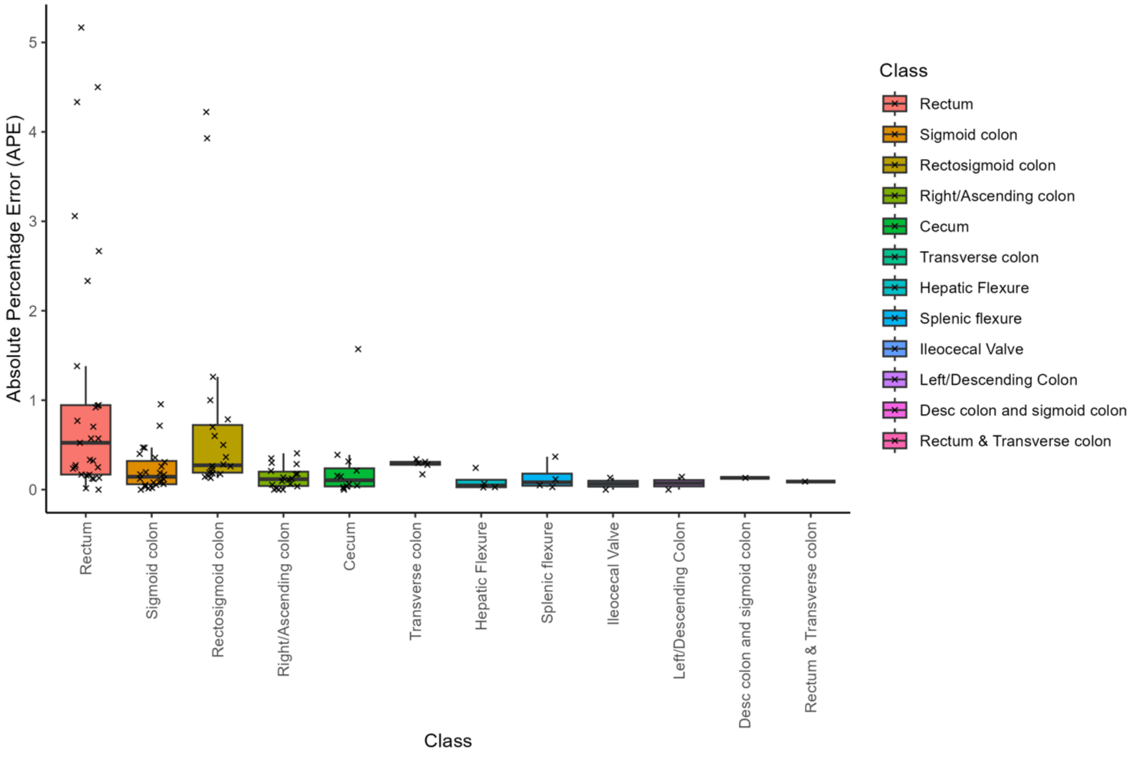


**Supplementary Figure 12.** Examining absolute percentage error by tumor specimen location.

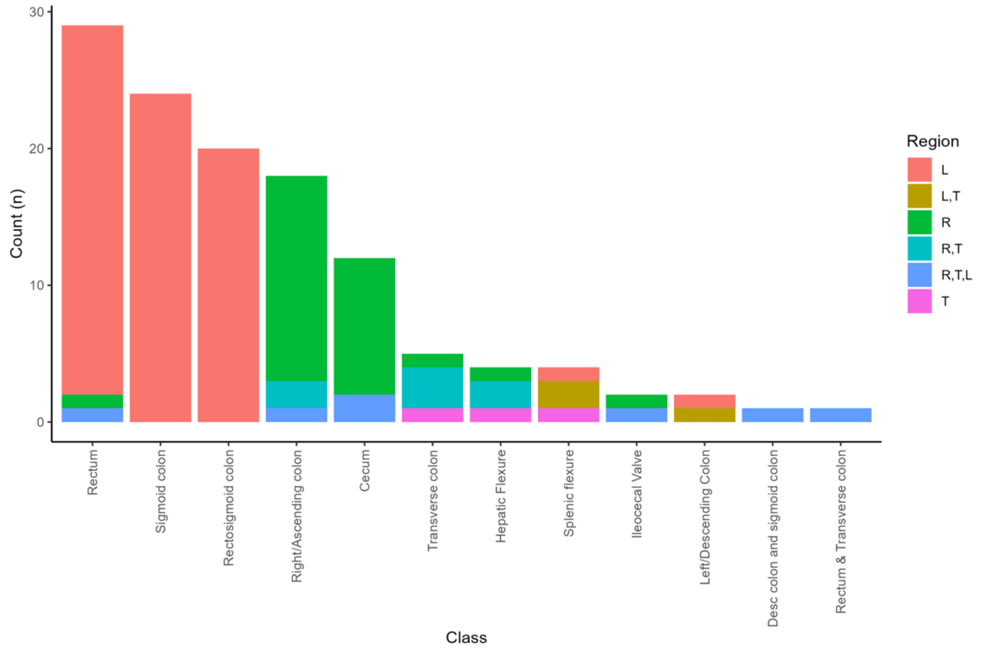


**Supplementary Figure 13.** Distribution of colorectal cancer locations (e.g., rectum, sigmoid colon, right/ascending colon, etc.) mapped to six broader anatomical regions—Left (L), Left-Transverse (L,T), Right (R), Transverse (T), Right–Transverse (R,T), and Right–Transverse–Left (R,T,L). This visualization illustrates that location and region classifications often overlap but do not align perfectly.

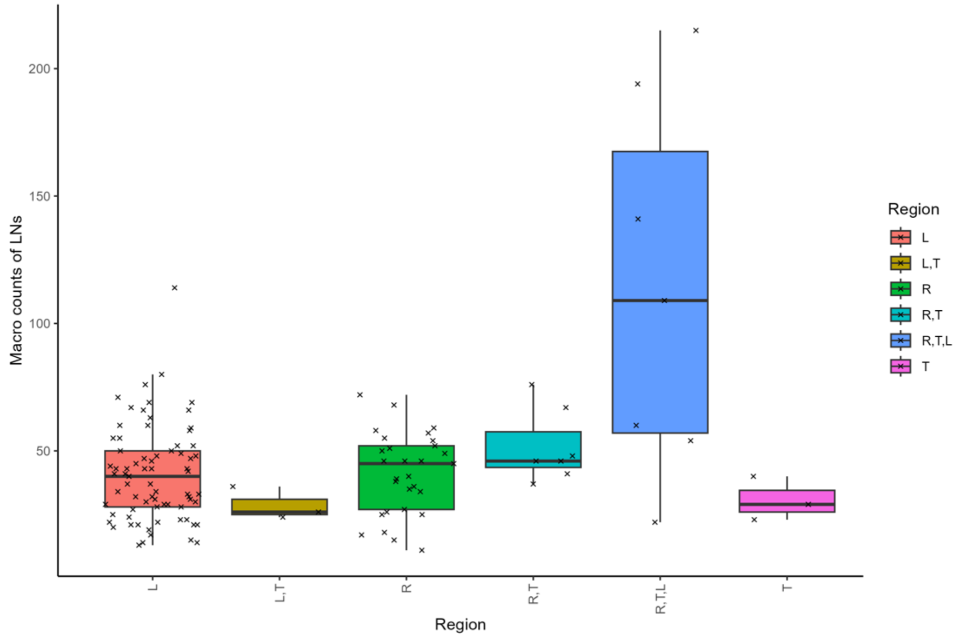


**Supplementary Figure 14.** Boxplot comparing macroscopic (gross) lymph node counts across the same six region categories (L, L-T, R, R-T, R-T-L, and T). The R,T,L group generally shows higher LN yields, while L and R largely overlap, indicating variable LN retrieval by region.

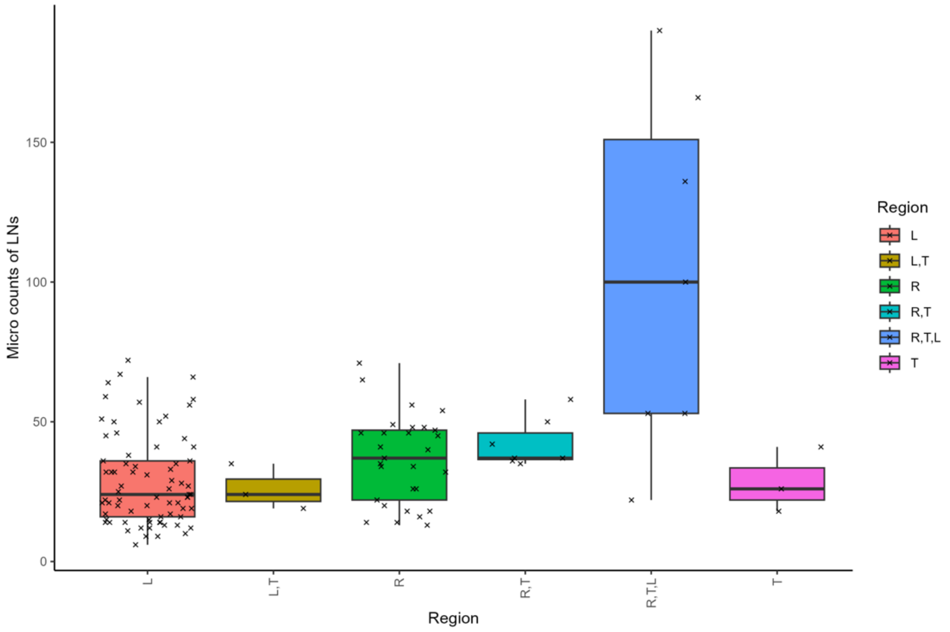


**Supplementary Figure 15.** Boxplot examining microscopic (histologically confirmed) lymph node counts for each region category. The trend is similar to macroscopic findings, but the difference between L and R appears more pronounced when focusing on nodes confirmed under the microscope.

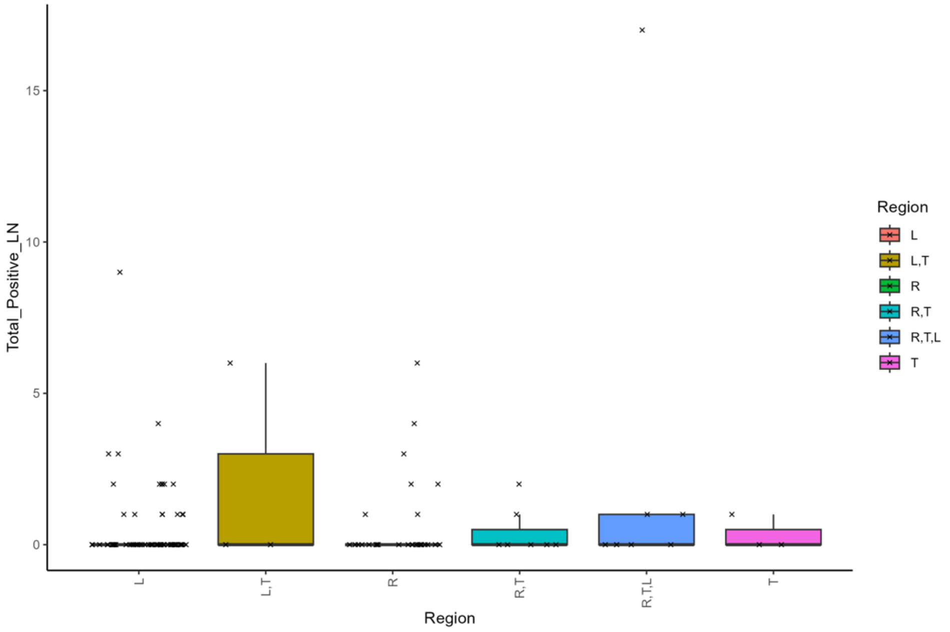


**Supplementary Figure 16.** Boxplot depicting cancer-positive lymph node counts by region, revealing no statistically significant differences in metastatic LN yield among the six region classifications, despite variations in total LN counts observed in Supplemental Figures 2 and 3.

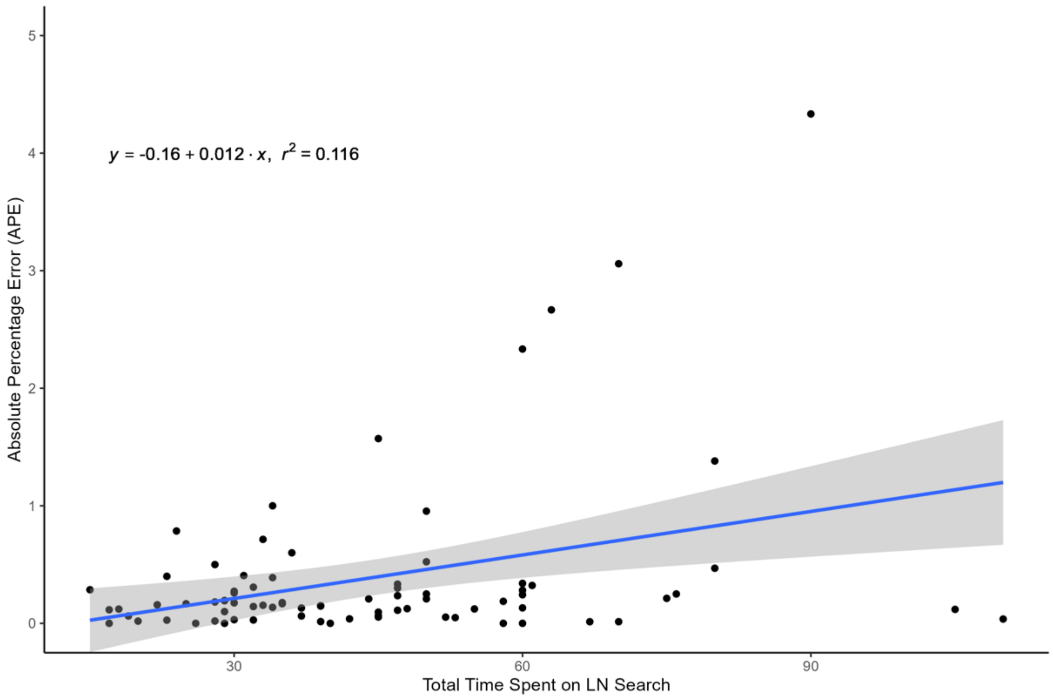


**Supplementary Figure 17.** Time spent on LN search process versus Absolute Percentage Error for single examiner samples from "rectum", “sigmoid colon", "rectosigmoid colon", "right/ascending colon", "cecum", "transverse colon", "hepatic flexure", and "splenic flexure".

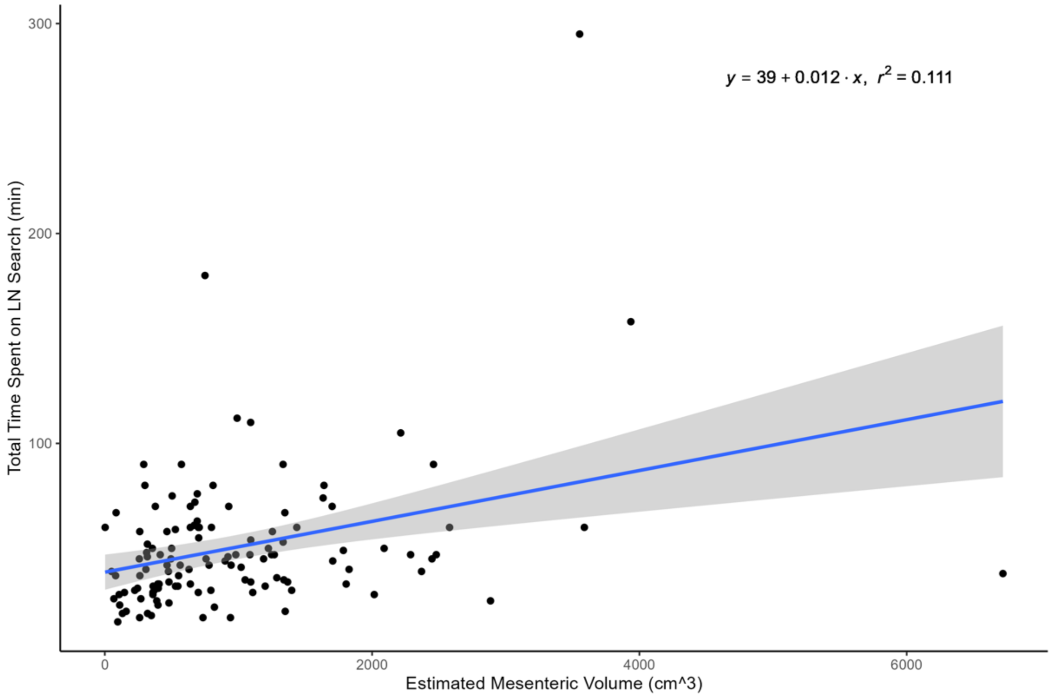


**Supplementary Figure 18.** Mesocolon tissue volume and its correlation with total search time for all samples.

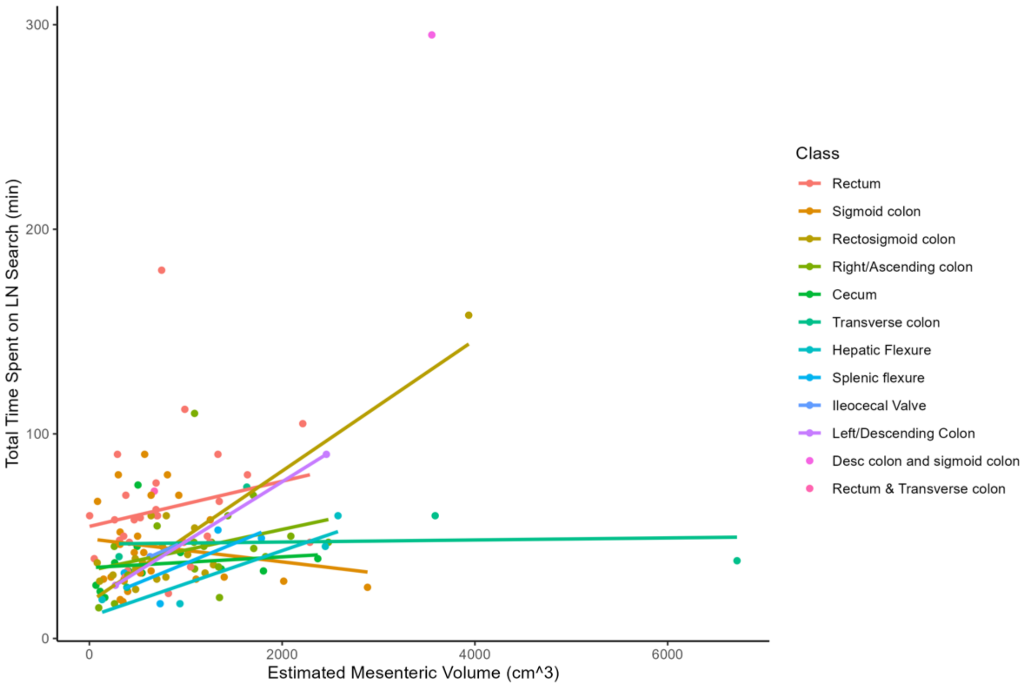


**Supplementary Figure 19.** Mesocolon tissue volume and its correlation with total search time for samples separated by tumor specimen location.

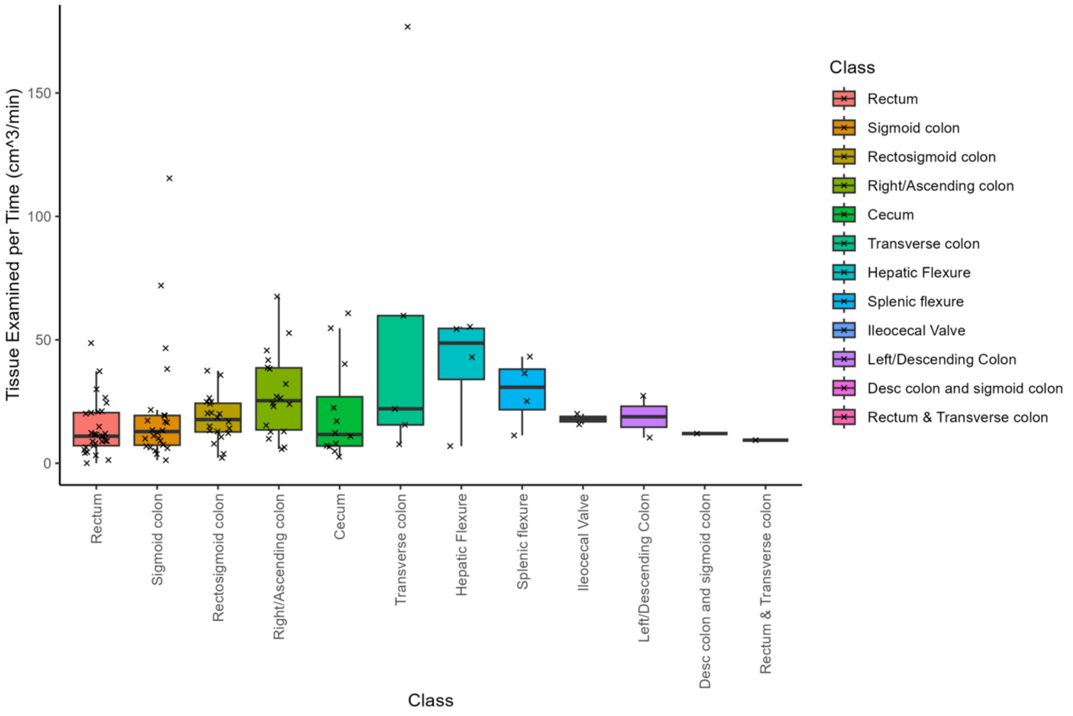


**Supplementary Figure 20.** Tissue examined per time ($cm^3$/min) versus tumor specimen location.

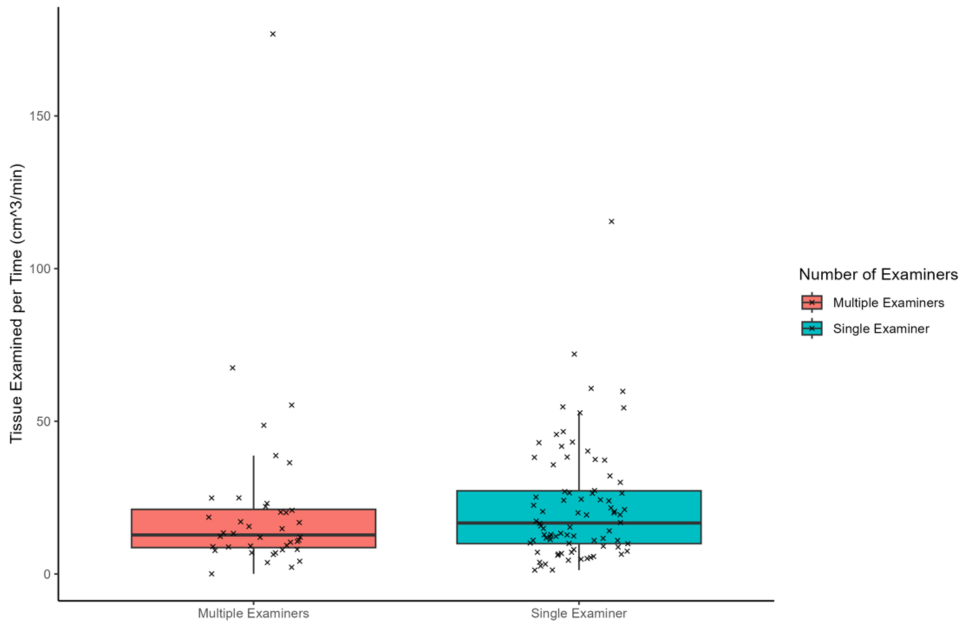


**Supplementary Figure 21.** Tissue examined per time ($cm^3$/min) versus presence of multiple examiners for a sample.

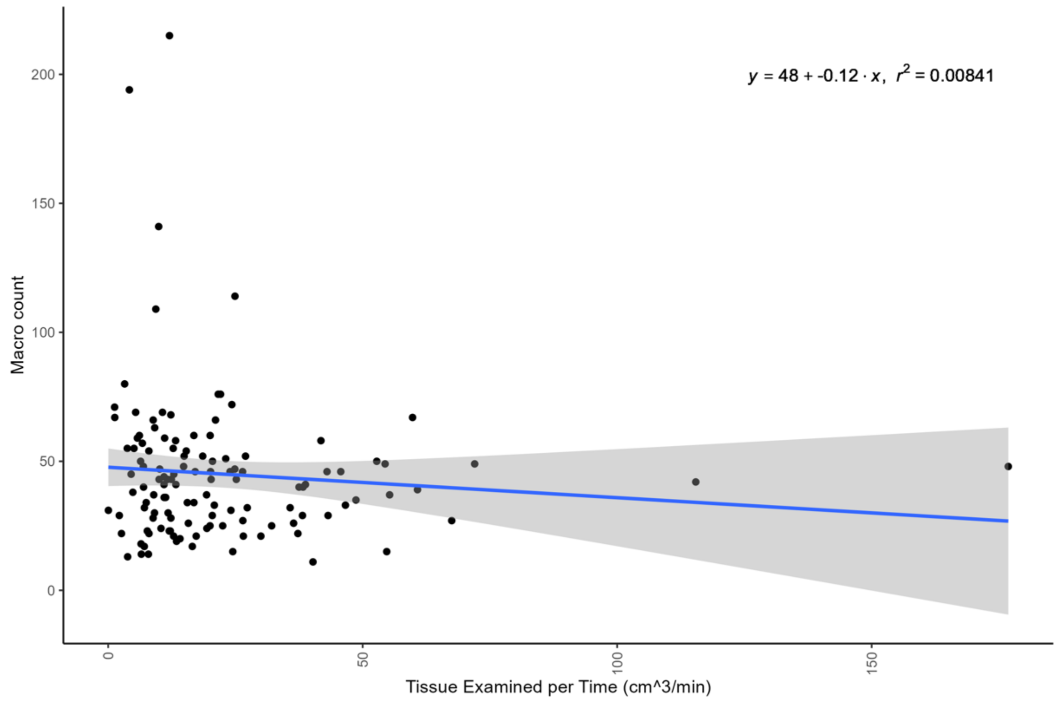


**Supplementary Figure 22.** Examining correlation between tissue examined per time ($cm^3$/min) and gross/macro lymph node counts for all samples.

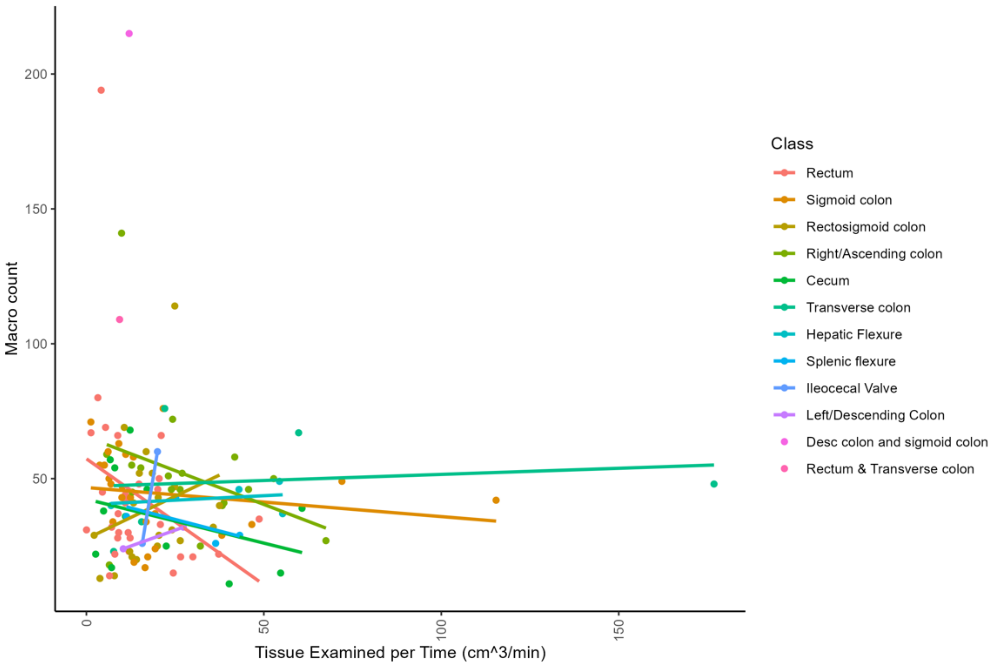


**Supplementary Figure 23.** Tissue examined per time (cm$^3$/min) versus gross LN counts for samples separated by tumor specimen location.

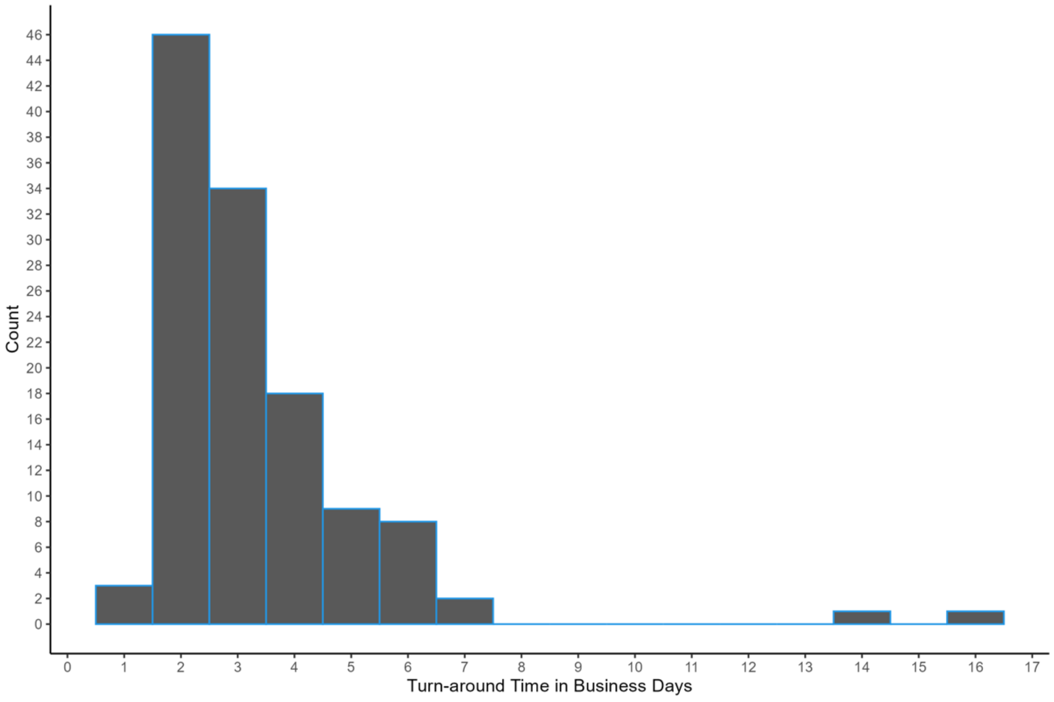


**Supplementary Figure 24.** Average turnaround time for pathology reports (in business days).

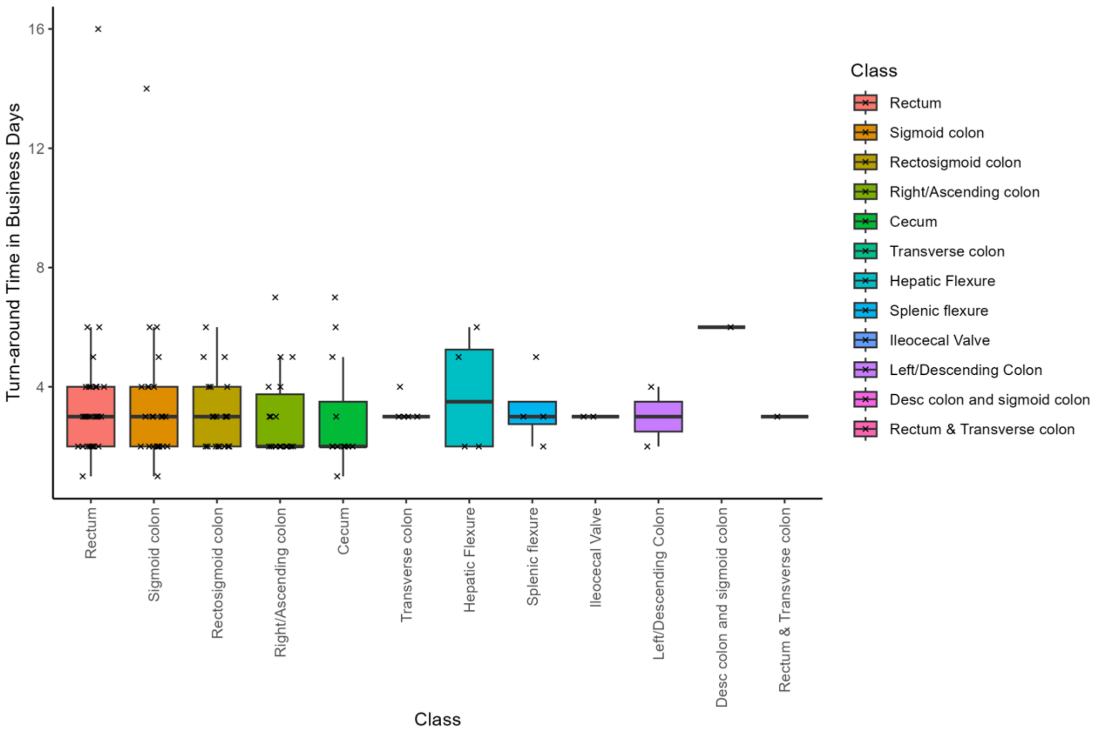


**Supplementary Figure 25.** Impact of cancer locations on average turnaround time for pathology report.

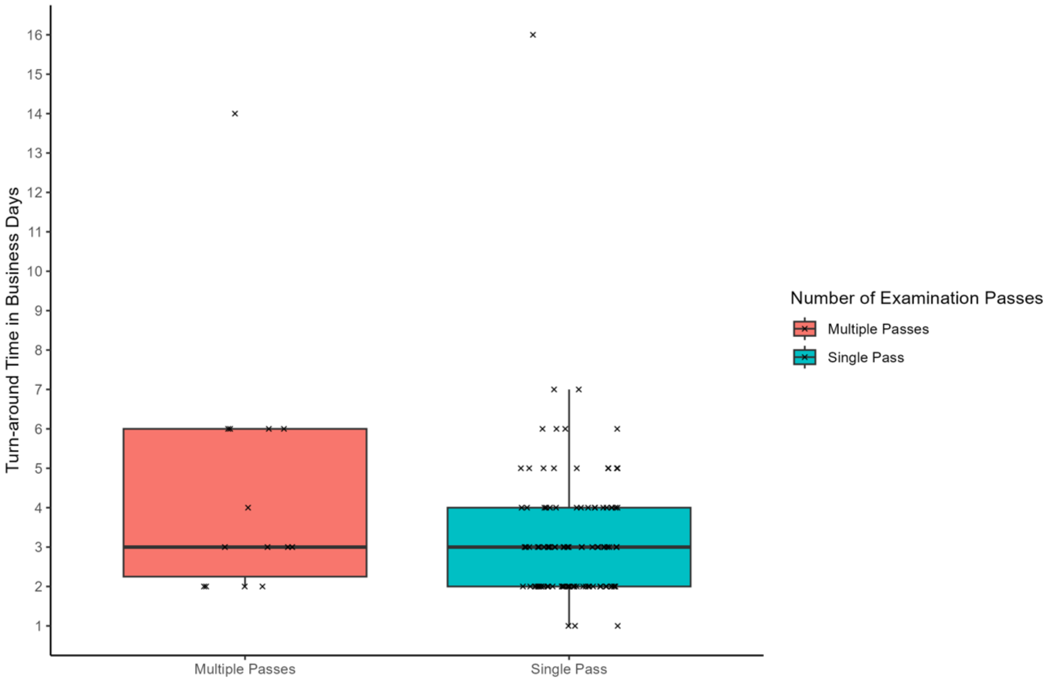


**Supplementary Figure 26.** Secondary LN searches and impact on average TAT for pathology report.

# 2 Supplementary Tables

**Supplementary Table 1.** Kruskal-Wallis rank sum test of time of first pass by type. Chi-squared = 22.005, df=7, p-value = 0.003. Pairwise comparisons using Wilcoxon rank sum test with continuity correction. P-value adjusted using Benjamin-Hochberg method.

| | Rectum | Sigmoid colon | Rectosigmoid colon | Right/ascending colon | Cecum | Transverse colon | Hepatic flexure |
|---|---|---|---|---|---|---|---|
| Sigmoid colon | 0.047 | - | - | - | - | - | - |
| Rectosigmoid colon | 0.007 | 0.943 | - | - | - | - | - |
| Right/ascending colon | 0.075 | 0.943 | 0.485 | - | - | - | - |
| Cecum | 0.008 | 0.943 | 0.943 | 0.360 | - | - | - |
| Transverse colon | 0.242 | 1.000 | 0.943 | 0.943 | 0.943 | - | - |
| Hepatic flexure | 0.242 | 0.943 | 0.943 | 0.943 | 0.943 | 0.943 | - |
| Splenic flexure | 0.242 | 0.943 | 0.973 | 0.943 | 0.943 | 0.972 | 0.943 |

**Supplementary Table 2.** Kruskal-Wallis rank sum test of total sample time by type. Chi-squared = 20.166, df=7, p-value = 0.005. Pairwise comparisons using Wilcoxon rank sum test with continuity correction. P-value adjusted using Benjamin-Hochberg method.

| | Rectum | Sigmoid colon | Rectosigmoid colon | Right/ascending colon | Cecum | Transverse colon | Hepatic flexure |
|---|---|---|---|---|---|---|---|
| Sigmoid colon | 0.058 | - | - | - | - | - | - |

| | | | | | | | |
|---|---|---|---|---|---|---|---|
| Rectosigmoid colon | 0.009 | 1.0000 | - | - | - | - | - |
| Right/ascending colon | 0.058 | 0.984 | 0.856 | - | - | - | - |
| Cecum | 0.009 | 0.917 | 0.917 | 0.448 | - | - | - |
| Transverse colon | 0.834 | 0.984 | 0.917 | 0.994 | 0.834 | - | - |
| Hepatic flexure | 0.261 | 0.910 | 0.984 | 0.917 | 0.984 | 0.917 | - |
| Splenic flexure | 0.300 | 0.984 | 1.0000 | 0.984 | 1.0000 | 0.917 | 1.0000 |

**Supplementary Table 3.** Kruskal-Wallis rank sum test of micro LN counts by tissue type. Chi-squared = 25.524, df = 7, p-value = 0.001. Pairwise comparisons using Wilcoxon rank sum test with continuity correction. P-value adjusted using Benjamin-Hochberg method.

| | Rectum | Sigmoid colon | Rectosigmoid colon | Right/ascending colon | Cecum | Transverse colon | Hepatic flexure |
|---|---|---|---|---|---|---|---|
| Sigmoid colon | 0.019 | - | - | - | - | - | - |
| Rectosigmoid colon | 0.647 | 0.062 | - | - | - | - | - |
| Right/ascending colon | 0.009 | 0.584 | 0.017 | - | - | - | - |
| Cecum | 0.316 | 0.488 | 0.488 | 0.223 | - | - | - |
| Transverse colon | 0.183 | 0.671 | 0.214 | 0.970 | 0.420 | - | - |
| Hepatic flexure | 0.118 | 0.671 | 0.118 | 0.671 | 0.420 | 0.766 | - |
| Splenic flexure | 0.316 | 0.584 | 0.488 | 0.295 | 0.887 | 0.488 | 0.316 |

**Supplementary Table 4.** Median and MAD scores of LN counts at the macro and micro level across different tissue types.

| Class | Med_Gross | MAD_Gross | Med_Micro | MAD_Micro |
|---|---|---|---|---|
| Hepatic flexure | 43.0 | 6.6717 | 39.0 | 3.7065 |
| Rectum | 35.0 | 17.7912 | 19.0 | 7.4130 |
| Splenic flexure | 32.5 | 7.4130 | 30.5 | 11.1195 |
| Rectosigmoid colon | 31.5 | 16.3086 | 23.0 | 13.3434 |
| Transverse colon | 48.0 | 28.1694 | 41.0 | 13.3434 |
| Right/Ascending colon | 48.0 | 10.3782 | 45.5 | 14.0847 |
| Cecum | 37.0 | 23.7216 | 30.0 | 19.2738 |
| Sigmoid colon | 45.0 | 17.0499 | 35.0 | 22.2390 |

**Supplementary Table 5.** ANOVA of T-stage versus microscopic LN counts.

| | df | Sum Sq | Mean Sq | F-value | P-value |
|---|---|---|---|---|---|
| T-stage | 7 | 11757 | 1679.6 | 2.55 | 0.018 |
| Residuals | 105 | 69167 | 658.7 | | |

**Supplementary Table 6.** ANOVA of T-stage versus cancer-positive LN counts.

| | df | Sum Sq | Mean Sq | F-value | P-value |
|---|---|---|---|---|---|
| T-stage | 7 | 46.1 | 6.592 | 1.579 | 0.150 |

| | | | | | |
|---|---|---|---|---|---|
| Residuals | 105 | 438.4 | 4.175 | | |

**Supplementary Table 7.** Factors impacting macroscopic LN counts.

| | Df | Sum Sq | Mean Sq | F value | Pr(>F) | |
|---|---|---|---|---|---|---|
| Class | 7 | 5336 | 762 | 5.241 | 1.44E-4 | *** |
| NeoAdjuvant | 2 | 609 | 304 | 2.092 | 0.134 | |
| Total_Sample_Time | 1 | 12125 | 12125 | 83.352 | 2.19E-12 | *** |
| Tstage | 7 | 4219 | 603 | 4.143 | 0.001 | ** |
| mesocolonTissueVolume | 1 | 24 | 24 | 0.162 | 0.689 | |
| TumorPresent | 1 | 4 | 4 | 0.03 | 0.862 | |
| singleExaminer_years | 2 | 68 | 34 | 0.235 | 0.791 | |
| Residuals | 52 | 7564 | 145 | | | |
| --- | | | | | | |
| Signif. codes:  0 ‘***’ 0.001 ‘**’ 0.01 ‘*’ 0.05 ‘.’ 0.1 ‘ ’ 1 | | | | | | |
| 5 observations deleted due to missingness | | | | | | |

**Supplementary Table 8.** Factors impacting microscopic LN counts.

| | Df | Sum Sq | Mean Sq | F value | Pr(>F) | |
|---|---|---|---|---|---|---|

| | | | | | | |
|---|---|---|---|---|---|---|
| Class | 7 | 6158 | 880 | 9.138 | 2.89E-07 | *** |
| NeoAdjuvant | 2 | 619 | 310 | 3.217 | 0.048 | * |
| Total_Sample_Time | 1 | 6498 | 6498 | 67.499 | 6.69E-11 | *** |
| Tstage | 7 | 5610 | 801 | 8.324 | 9.58E-07 | *** |
| mesocolonTissueVolume | 1 | 77 | 77 | 0.798 | 0.376 | |
| TumorPresent | 1 | 46 | 46 | 0.481 | 0.491 | |
| singleExaminer_years | 2 | 377 | 189 | 1.959 | 0.152 | |
| Total_Macro_Ln | 1 | 4565 | 4565 | 47.413 | 8.25E-09 | *** |
| Residuals | 51 | 4910 | 96 | | | |
| --- | | | | | | |
| Signif. codes:  0 ‘***’ 0.001 ‘**’ 0.01 ‘*’ 0.05 ‘.’ 0.1 ‘ ’ 1 | | | | | | |
| 5 observations deleted due to missingness | | | | | | |

**Supplementary Table 9.** Factors impacting total positive LN microscopic counts.

| | Df | Sum Sq | Mean Sq | F value | Pr(>F) | |
|---|---|---|---|---|---|---|
| Class | 10 | 17.6 | 1.76 | 0.911 | 0.527 | |
| NeoAdjuvant | 1 | 3.23 | 3.234 | 1.674 | 0.199 | |
| Total_Sample_Time | 1 | 0.26 | 0.256 | 0.133 | 0.717 | |

| | | | | | | |
|---|---|---|---|---|---|---|
| Tstage | 7 | 26.89 | 3.842 | 1.988 | 0.066 | . |
| mesocolonTissueVolume | 1 | 0.03 | 0.026 | 0.014 | 0.907 | |
| Total_Macro_Ln | 1 | 0.03 | 0.03 | 0.015 | 0.901 | |
| Total_Micro_Ln | 1 | 0.06 | 0.064 | 0.033 | 0.856 | |
| Residuals | 88 | 170.01 | 1.932 | | | |
| --- | | | | | | |
| Signif. codes:  0 ‘***’ 0.001 ‘**’ 0.01 ‘*’ 0.05 ‘.’ 0.1 ‘ ’ 1 | | | | | | |
| 5 observations deleted due to missingness | | | | | | |

**Supplementary Table 10.** Differences between the size of the largest LN and sample classes.

| | Rectum | Sigmoid colon | Rectosigmoid colon | Right/ascending colon | Cecum | Transverse colon | Hepatic flexure |
|---|---|---|---|---|---|---|---|
| Sigmoid colon | 0.823 | - | - | - | - | - | - |
| Rectosigmoid colon | 0.891 | 0.976 | - | - | - | - | - |
| Right/Ascending colon | 0.031 | 0.031 | 0.031 | - | - | - | - |
| Cecum | 0.028 | 0.028 | 0.028 | 0.472 | - | - | - |
| Transverse colon | 0.349 | 0.349 | 0.349 | 0.891 | 0.472 | - | - |
| Hepatic Flexure | 0.095 | 0.095 | 0.031 | 0.891 | 0.814 | 0.970 | - |
| Splenic flexure | 0.472 | 0.527 | 0.472 | 0.893 | 0.668 | 1.000 | 0.794 |

P value adjustment method: BH

**Supplementary Table 11.** MAPE scores for groups based on "SingleGrosser" samples.

| | AllSingleGrosser | < 1 year | 1-5 years | 6 or more |
|---|---|---|---|---|
| MAPE | 0.375127 | 0.446653 | 0.513182 | 0.210647 |
| sdApe | 0.702117 | 0.753933 | 0.929385 | 0.261526 |
| medianAPE | 0.162281 | 0.1875 | 0.164439 | 0.157895 |
| MAD_APE | 0.17233 | 0.129728 | 0.183289 | 0.170251 |

**Supplementary Table 12.** Mean macro and micro-LN counts broken down by examiner experience.

| | < 1 year | 1-5 years | 6 or more |
|---|---|---|---|
| mean_Gross | 35 | 38.55556 | 44.31429 |
| mean_Micro | 17.83153 | 15.84769 | 22.38615 |

**Supplementary Table 13.** Pairwise comparisons using Wilcoxon rank sum test of APE by Sample Class.

| | Rectum | Sigmoid colon | Rectosigmoid colon | Right/Ascending colon | Cecum | Transverse colon | Hepatic Flexure |
|---|---|---|---|---|---|---|---|
| Sigmoid colon | 0.016 | - | - | - | - | - | - |
| Rectosigmoid colon | 0.753 | 0.022 | - | - | - | - | - |

| | | | | | | | |
|---|---|---|---|---|---|---|---|
| Right/Ascending colon | 0.006 | 0.571 | 0.006 | - | - | - | - |
| Cecum | 0.033 | 0.659 | 0.045 | 0.914 | - | - | - |
| Transverse colon | 0.661 | 0.368 | 0.910 | 0.112 | 0.272 | - | - |
| Hepatic Flexure | 0.067 | 0.419 | 0.059 | 0.738 | 0.783 | 0.098 | - |
| Splenic flexure | 0.098 | 0.659 | 0.098 | 1.000 | 1.000 | 0.473 | 0.658 |
| P value adjustment method: BH | | | | | | | |

**Supplementary Table 14.** ANOVA model examining several variables using the Absolute Percentage Error calculated between macro and micro-LN counts.

| | Df | Sum Sq | Mean Sq | F value | Pr(>F) | |
|---|---|---|---|---|---|---|
| Class | 7 | 7.517 | 1.0738 | 2.24 | 0.045 | * |
| NeoAdjuvant | 2 | 0.018 | 0.009 | 0.019 | 0.981 | |
| Total_Sample_Time | 1 | 1.821 | 1.8211 | 3.799 | 0.057 | . |
| Tstage | 7 | 1.582 | 0.226 | 0.472 | 0.851 | |
| mesocolonTissueVolume | 1 | 0.674 | 0.6739 | 1.406 | 0.241 | |
| singleExaminer_years | 2 | 0.649 | 0.3247 | 0.677 | 0.512 | |
| Residuals | 53 | 25.405 | 0.4793 | | | |
| --- | | | | | | |
| Signif. codes: 0 '***' 0.001 '**' 0.01 '*' 0.05 '.' 0.1 ' ' 1 | | | | | | |

| | | | |
|---|---|---|---|
| 5 observations deleted due to missingness | | | |

**Supplementary Table 15.** Rate of tissue examination over all samples (rate = volume/time).

| | |
|---|---|
| Mean tissue examination rate (cm^3/min) | 21.94 |
| Standard deviation of tissue examination rate | 22.62 |
| Median tissue examination rate (cm^3/min) | 15.13 |
| Mean Absolute Deviation of tissue examination rate | 11.85 |
| Minimum tissue examination rate (cm^3/min) | 0.04 |
| Maximum tissue examination rate (cm^3/min) | 176.84 |

**Supplementary Table 16.** Kruskal-Wallis rank sum test of Total Micro LN counts by Region. Chi-squared = 14.2, df = 5, p-value = 0.0144. Pairwise comparisons using Wilcoxon rank sum test. Pairwise comparisons using Wilcoxon rank sum test with continuity correction. Legend = L: Left colon; L,T: Left, transverse colon; R: Right colon; R,T: Right, transverse colon; and R,T,L: Right, transverse and left colon.

| | L | L,T | R | R,T | R,T,L |
|---|---|---|---|---|---|
| L,T | 1.000 | - | - | - | - |
| R | 0.130 | 0.570 | - | - | - |
| R,T | 0.130 | 0.230 | 0.560 | - | - |
| R,T,L | 0.130 | 0.460 | 0.230 | 0.500 | - |
| T | 0.920 | 1.000 | 0.560 | 0.430 | 0.460 |
| P value adjustment method: BH | | | | | |

**Supplementary Table 17.** Kruskal-Wallis rank sum test of Total Macro LN counts by Region. Chi-squared = 7.4357, df = 5, p-value = 0.190. Pairwise comparisons using Wilcoxon rank sum test with

continuity correction. Legend = L: Left colon; L,T: Left, transverse colon; R: Right colon; R,T: Right, transverse colon; and R,T,L: Right, transverse and left colon.

| | L | L,T | R | R,T | R,T,L |
|---|---|---|---|---|---|
| L,T | 0.560 | - | - | - | - |
| R | 0.570 | 0.540 | - | - | - |
| R,T | 0.440 | 0.420 | 0.540 | - | - |
| R,T,L | 0.440 | 0.560 | 0.460 | 0.540 | - |
| T | 0.540 | 1.000 | 0.540 | 0.420 | 0.540 |
| P value adjustment method: BH | | | | | |

**Supplementary Table 18.** Kruskal-Wallis rank sum test of Total Cancer Positive LN counts by Region. Chi- squared = 5.894, df = 5, p-value = 0.317. Legend = L: Left colon; L,T: Left, transverse colon; R: Right colon; R,T: Right, transverse colon; and R,T,L: Right, transverse and left colon.

| | L | L,T | R | R,T | R,T,L |
|---|---|---|---|---|---|
| L,T | 0.850 | - | - | - | - |
| R | 1.000 | 0.850 | - | - | - |
| R,T | 1.000 | 1.000 | 1.000 | - | - |
| R,T,L | 0.450 | 1.000 | 0.530 | 0.850 | - |
| T | 1.000 | 1.000 | 1.000 | 1.000 | 0.850 |
| P value adjustment method: BH | | | | | |
